# Aromatic Wall Extension of Glycoluril‐Derived Molecular Clips Enhances Binding of Planar Aromatic Dyes

**DOI:** 10.1002/chem.202502177

**Published:** 2025-08-18

**Authors:** Collin J. Vincent, David King, Steven L. Murkli, Lyle Isaacs

**Affiliations:** ^1^ Department of Chemistry and Biochemistry University of Maryland College Park College Park Maryland 20742 USA

**Keywords:** dyes, glycoluril, molecular clip, p‐extension, spectral titration

## Abstract

We report the synthesis and characterization of a new methylene‐bridged glycoluril dimer featuring anthracene walls (**H2**). **H2** displays good solubility in water (≥7 mM) but undergoes self‐association at concentrations above 2 mM. ^1^H NMR experiments establish that **H2** binds cationic dyes inside its cavity with a π‐stacked geometry that places the cationic residues at the ureidyl carbonyl portals of **H2**. The binding constants of both naphthalene‐walled clip **H1** and anthracene‐walled clip **H2** toward a panel of dyes were measured by direct or competitive UV/Vis or fluorescence titrations in phosphate buffered saline (PBS). Binding constants cover the range from 10^3^ – 10^8^ M^−1^. Dyes that feature cationic NMe_2_ groups bind more strongly than analogous dyes with cationic NH_2_ groups. We find that π‐extension of the aromatic walls from **H1** to **H2** generally results in an ≈ 10‐fold increase in binding affinity. Host•guest complexes of **H1** and **H2** with planar cationic dyes benefit from substantial cation‐π interactions.

## Graphical abstract

The influence of the length of the aromatic walls of methylene‐bridged glycoluril‐dimer‐derived molecular clips on their binding affinity toward a panel of planar aromatic cations is reported. We find that anthracene‐walled host **H2** generally binds ≈ 10‐fold stronger to a specific guest than naphthalene‐walled **H1**.

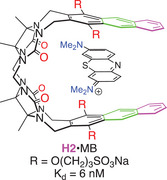

## 1 Introduction

**Figure 1:**
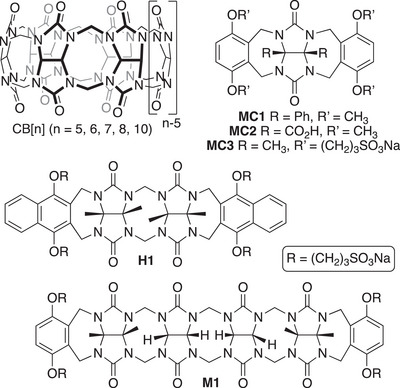
Chemical structures of selected glycoluril‐derived hosts.

Glycoluril‐derived supramolecular objects have played a starring role in the development of supramolecular chemistry, beginning with the inspirational work of Mock in the 1980s on cucurbit[6]uril.^[^ ^1^ ^]^ In the intervening time, the supramolecular chemistry of macrocyclic cucurbit[n]uril (Figure 1; CB[n], n = 5, 6, 7, 8, 10) unfolded rapidly following the discovery of CB[n] homologues (n = 5, 7, 8, 10) around the turn of the millennium.^[^ ^2^ ^]^ The ultratight binding affinity, high selectivity, and stimuli responsiveness of CB[n]•guest complexes have enabled numerous applications, including supramolecular latching, sensing ensembles, functional materials, and a variety of pharmaceutical and medicinal applications.^[^ ^3^ ^]^

**Scheme 1:**
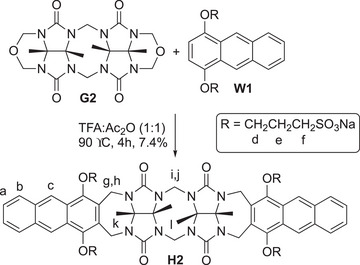
Synthesis of anthracene‐walled glycoluril dimer **H2**.

Acyclic glycoluril‐derived hosts have also been developed. For example, Rebek's group utilized the H‐bonding potential of the glycoluril urea functional groups to create self‐assembled dimers reminiscent of the shape of tennis balls and jelly doughnuts.^[^ ^4^ ^]^ Conversely, the Nolte group took advantage of the reactivity of diphenyl glycoluril bis(cyclic ether)s to install aromatic sidewalls, which resulted in the formation of glycoluril molecular clips (e.g., **MC1**), which form the basis of a variety of functional architectures, including processive catalysts.^[^ ^5^ ^]^ Water‐soluble versions of Nolte's diaryl glycoluril molecular clips display a tendency toward self‐association in water to create razor‐blade‐like objects.^[^ ^6^ ^]^ Our group prepared molecular clips based on glycoluril monomer (e.g., **MC2**) and methylene‐bridged glycoluril dimer bearing CO_2_H solubilizing groups on their convex face, which results in the formation of discrete dimeric assemblies in water.^[^ ^7^ ^]^ Later on, we progressed to acyclic CB[n]‐type receptors based on the full series of glycoluril oligomers (monomer, dimer, trimer, tetramer, pentamer).^[^ ^7^, ^8^ ^]^ We found that attachment of the solubilizing groups to the aromatic walls (e.g., **MC3**) rather than the convex face (e.g., **MC2**) largely suppresses the tendency toward self‐association and enables host•guest recognition properties. For example, we found that glycoluril tetramer‐derived **M1** and congeners function as a solubilizing excipients for insoluble drugs and as in vivo reversal agents for drugs of abuse, neuromuscular blocking agents, and anesthetics.^[^ ^9^ ^]^ As part of this line of inquiry, we also studied the influence of the nature of the aromatic walls (e.g., benzene, dialkyl benzene, naphthalene, anthracene, triptycene) on the host•guest binding affinity.^[^ ^10^ ^]^ Anthracene‐walled hosts have been extensively studied by Yoshizawa and co‐workers.^[^ ^11^ ^]^ We find that glycoluril tetramer‐derived hosts bind most tightly to hydrophobic alkyl ammonium ions. Most recently, we prepared **H1** (Figure 1), which features a glycoluril dimer backbone and roughly parallel naphthalene sidewalls, and showed that **H1** is selective for aromatic cations — particularly acridine‐derived dyes like methylene blue (**MB**) — over alkyl ammonium ions.^[^ ^12^ ^]^ **H1** is a powerful enough host to destain U87 cells that had previously been stained with **MB**. In this paper, we explore the impact of π‐extension of **H1** to the corresponding anthracene‐walled host **H2** (Scheme 1) on the host•guest binding affinity.

## 2 Results and Discussion

This results and discussion section is organized as follows. First, we describe the design, synthesis, and characterization of anthracene‐walled molecular clip **H2**. Next, we discuss the selection of the panel of guests (dyes) to be studied. Third, we detail the molecular recognition processes of homologous hosts **H1** and **H2** toward the guest panel by a combination of direct and competitive UV/Vis and fluorescence measurements along with ^1^H NMR spectroscopy. Finally, we discuss the trends seen in the binding constants as a function of host structure and guest structure.

### 2.1 Design, Synthesis, and Characterization of Anthracene‐Walled H2

**Figure 2:**
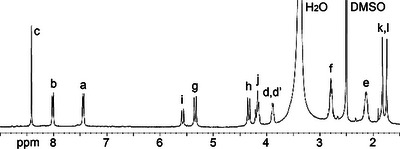
^1^H NMR spectrum recorded (600 MHz, DMSO‐*d*_6_, RT) for **H2**.

The design of **H2** is based on the premise that π‐extension of the walls of methylene‐bridged glycoluril dimers from **H1** to **H2** will increase the size of the hydrophobic cavity and thereby increase the binding affinity and selectivity toward planar aromatic guests.^[^ ^10c^ ^]^ The synthesis of glycoluril‐derived molecular clips and acyclic CB[n]‐type receptors follows a well‐trodden path involving the double electrophilic aromatic substitution reaction of a glycoluril oligomer bis(cyclic ether) building block with an aromatic wall building block.^[^ ^8b^ ^]^ Accordingly, to prepare anthracene‐walled molecular clip **H2**, we reacted **G2** with **W1** under hot acidic conditions (TFA, Ac_2_O, 90 °C) for 4 hours (Scheme 1). Although **H2** is a major product of this reaction, the isolation of pure **H2** was challenging and required extensive washing with acetone, re‐precipitation from water:acetone (1:20), gel permeation chromatography (Sephadex G25) to remove dark green colored impurities, along with a final washing with acetonitrile. Pure **H2** was obtained in 7.4% yield as a pale yellow‐green colored solid. **H2** is reasonably soluble (≥7 mM) in water. The spectral data recorded for **H2** are in accord with the *C*_2_ *_v_*‐symmetric structure depicted in Scheme 1. For example, the ^1^H NMR spectrum recorded in DMSO‐*d*_6_ (Figure 2) displays three resonances for the anthracene protons (H_a_ – H_c_), four resonances for the two different diastereotopic CH_2_‐groups (H_g_ – H_j_) in the expected 1:2 ratio, the resonances for the OCH_2_CH_2_CH_2_SO_3_Na arms, and two CH_3_‐groups (H_k_ – H_l_). Please note the appearance of the diastereotopic H_d_ and H_d’_ resonances at ≈ 4 ppm in DMSO. The ^13^C spectrum recorded in D_2_O exhibits the 17 expected resonances, including one for the C = O group, seven resonances for the anthracene walls, and two for the methyl groups (Figure S3). The electrospray ionization mass spectrum shows major ions at *m/z* 659 and 681, which correspond to the [**H2** – 4Na + 2H]^2−^ and [**H2** – 2Na]^2−^ species. The quantum yield of **H2** was determined as 0.025 (Supporting Information Figure S38) using **ProF** as a standard of known quantum yield.^[^ ^13^ ^]^ We also synthesized naphthalene‐walled molecular clip **H1** as a comparator compound by the literature procedure.^[^ ^8a^ ^]^

### 2.2 Self‐Association Properties of H2

Before proceeding toward the measurement of **H2**•dye binding constants, we wanted to investigate the potential of **H2** for self‐association. Although the ^1^H NMR spectrum of **H2** in DMSO‐*d*_6_ is consistent with a monomeric *C*_2_ *_v_*‐symmetric structure, the corresponding spectrum recorded at 2.8 mM in D_2_O (Figure S5) displays additional resonances that arise from dimeric or higher‐order oligomeric structures. Analogous spectra recorded at lower concentrations of **H2** (500 µM, Figure S5) are consistent with monomeric **H2**. Related dilution experiments monitored by UV/Vis spectroscopy from [**H2**] = 0.1 to 60 µM are linear in accord with Beer's law, which further indicates that **H2** is monomeric over this concentration range, which will be used for the optical titrations reported below.

### 2.3 Selection of the Panel of Dyes for H1 and H2

**Figure 3:**
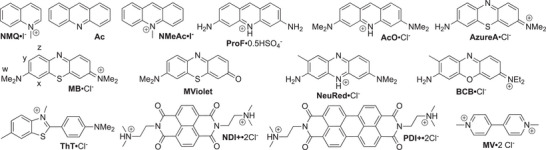
Chemical structures of dyes used in this study.

Previously, we have studied the molecular recognition behavior of **H1** toward a panel of 32 guests in 20 mM sodium phosphate buffered water.^[^ ^8a^ ^]^ Unlike macrocyclic CB[n], the cavity of **H1** is defined by two roughly co‐planar naphthalene rings. Accordingly, **H1** binds preferentially to planar aromatic cations compared to alkyl (di)ammonium ions, which are the best guests for macrocyclic CB[n]. The selection of the 14 dyes shown in Figure 3 as guests for this study was guided by several considerations. First, the anthracene walls of **H2** are UV/Vis active from 320 to 410 nm, which means that suitable guests must absorb outside this region. Second, both **H2** and many dyes are prone to aggregation at mM concentrations in water, which means that high‐affinity **H2**•dye complexes will be most straightforward to study at the required low µM concentrations. The selected dyes (Figure 3) fulfill these requirements. Finally, the selected dyes will allow us to tease out the importance of guest charge, guest methylation state, π‐surface area, and the cation‐π interactions on complexation.

### 2.4 Investigation of H2•Dye Complexation by ^1^H NMR Spectroscopy

**Figure 4:**
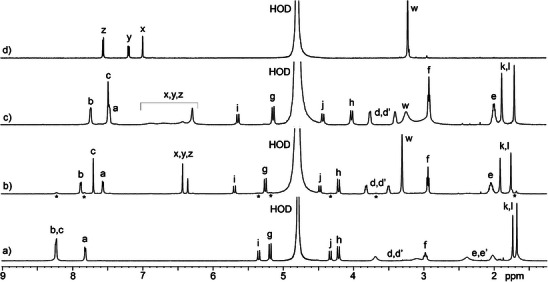
^1^H NMR spectra recorded (600 MHz, D_2_O, RT) for: a) **H2** (0.5 mM), b) a mixture of **H2** (0.5 mM) and **MB** (0.5 mM), c) a mixture of **H2** (0.5 mM) and **MB** (1.0 mM), and d) **MB** (0.5 mM). Resonance marked with an asterisk (*) arises from uncomplexed **H2**.

**Figure 5:**
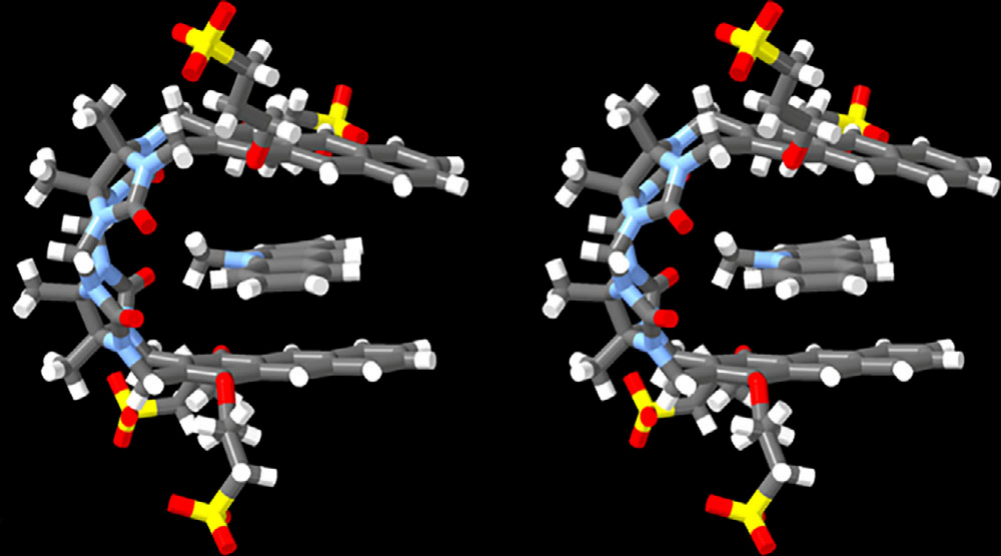
Cross‐eyed stereoview of the MMFF‐minimized geometry of **H2**•**NMeAc**. Color code: C, gray; H, white; N, blue; O, red; S, yellow.

Initially, we chose to explore the complexation between **H2** and dyes by ^1^H NMR spectroscopy. For this purpose, we selected the tighter binding and more symmetrical dyes **MB**, N‐methylacridinium (**NMeAc**), proflavin (**ProF**), and cationic naphthalene diimide (**NDI**+), which were expected to display more easily interpretable spectra. Figure 4 shows the ^1^H NMR spectra recorded for uncomplexed **H2**, uncomplexed **MB**, and 1:1 and 1:2 mixtures of **H2** and **MB**. Several features of the spectra are noteworthy. For example, the ^1^H NMR resonances for the aromatic resonances of **MB** (H_x_, H_y_, H_z_) undergo significant upfield shifts upon formation of **H2**•**MB** due to the anisotropic shielding effect of the anthracene walls of **H2**. Conversely, the NCH_3_‐group (H_w_) of **MB** (Figure 4d) undergoes a slight downfield shift upon complexation (Figure 4b), which is typical of protons located near the ureidyl carbonyl portals of glycoluril‐derived receptors.^[^ ^14^ ^]^ At a 1:2 **H2**:**MB** stoichiometry, the **MB** protons broaden and shift back toward the chemical shift of uncomplexed **MB,** which indicates intermediate exchange kinetics on the chemical shift timescale. Conversely, the aromatic protons of host **H2** (H_a_ – H_c_, Figure 4a) also undergo upfield shifts upon formation of the **H2**•**MB** complex which can be attributed to the anisotropic magnetic shielding of the **MB** guest on the **H2** protons. Interestingly, the resonance for H_i_ undergoes a 0.34 ppm downfield shift upon formation of **H2**•**MB,** which can be attributed to conformational changes that occur upon binding that bring H_i_ closer to the deshielding region near the C = O lone pairs of **H2**. Related complexation induced upfield shifts of the protons of **H2** and dye are also observed for the **H2**•**ProF**, **H2**•**NDI**+, and **H2**•**NMeAc** complexes (Figures S35‐37) which establish related π‐stacked geometries for these complexes. An interesting aspect of the ^1^H NMR spectrum of **H2**•**NMeAc** is the resonance for NCH_3_ at ≈ 3.32 ppm, which is shifted upfield from 4.76 ppm for uncomplexed **NMeAc**. This observation suggests that the NCH_3_‐group is oriented into the cavity. Figure 5 shows a cross‐eyed stereoview of an MMFF‐minimized molecular model of **H2**•**NMeAc** whose geometry is consistent with complexation‐induced changes in chemical shift of both **H2** and **NMeAc** noted above.

### 2.5 Measurement of Binding Constants between Hosts and Guests

**Figure 6:**
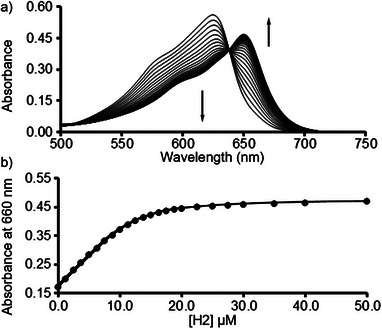
a) UV/Vis spectra recorded during the titration of **BCB** (10.0 µM) with **H2** (0 – 50.0 µM). b) Plot of A_650_ versus [**H2**]. The solid line represents the best fit of the data to a 1:1 binding model implemented in Scientist^TM^ with K_a_ = 8.27 ± 0.15 × 10^5^ M^−1^.

After qualitatively determining the geometry of selected **H2**•guest complexes by analysis of complexation‐induced changes in chemical shift, we turned to a measurement of the **H2**•dye binding constants along with those of **H1**•dye complexes as a comparator. We elected to use the biologically relevant PBS buffer as the solvent, which also reduces K_a_ values by competitive binding of alkali metal ions at the ureidyl C = O portals.^[^ ^15^ ^]^ As described above, both the dyes and **H2** are prone to aggregation at higher concentrations in water, which precluded binding constant determination by isothermal titration calorimetry whereas the large **H2**•dye K_a_ values (vide infra) precluded the use of ^1^H NMR titrations. Accordingly, we turned to UV/Vis and fluorescence spectroscopy as suitable analytical methods at the required low µM concentrations. For dyes whose UV/Vis λ_max_ is beyond the absorbance of the anthracene walls, direct UV/Vis titrations of fixed concentrations of dye with hosts **H1** or **H2** are appropriate. For example, Figure 6a shows the UV/Vis spectra recorded during the titration of brilliant cresyl blue (**BCB**, 10 µM) with a solution of **H2** (0 – 50 µM). The series of UV/Vis spectra show an isosbestic point at 639 nm, which is indicative of a well‐defined two‐state equilibrium. We observe a bathochromic shift from 599 nm for uncomplexed **BCB** to 649 nm for **H2**•**BCB**. Figure 6b shows a plot of absorbance at 650 nm versus [**H2**] which was fitted to a 1:1 binding model with K_a_ = 8.27 ± 0.15 × 10^5^ M^−1^ (Table 1) for **H2**•**BCB**. Analogous direct UV/Vis titrations were performed for the complexes of **H2** with methylene violet (**MViolet**), neutral red (**NeuRed**), and cationic perylene diimide (**PDI+**) and for **H1** with **Ac**, **NMeAc**, **AzureA**, **MB**, **MViolet**, **NeuRed**, **BCB**, **NDI+**, and **PDI +** which are presented in the Supporting Information and the K_a_ values are given in Table 1.

**Table 1:** Binding Constants (K_a_, M^−1^) measured by UV/Vis or fluorescence spectroscopy for the **H2**•dye (PBS, RT) and **H1**•dyes (PBS, RT and 20 mM sodium phosphate, RT) complexes. All titrations were performed in duplicate.

Guest	H2 [M^−1^] PBS	H1 [M^−1^] PBS	H1 [M^−1^] 20 mM phosphate^[^ ^12^ ^]^
**NMeQ**	(1.50 ± 0.05) × 10^4[^ ^c^ ^]^	(6.34 ± 0.08) × 10^3[^ ^e^ ^]^	8.03 ± 0.6 × 10^3^
**Ac**	(5.26 ± 0.30) × 10^5[^ ^d^ ^]^	(8.31 ± 0.12) × 10^3[^ ^a^ ^]^	1.09 ± 0.09 × 10^4^
**NMeAc**	(1.07 ± 0.10) × 10^7[^ ^d^ ^]^	(2.59 ± 0.36) × 10^5[^ ^a^ ^]^	5.07 ± 0.6 × 10^5^
**ProF**	(2.78 ± 0.06) × 10^6[^ ^b^ ^]^	(2.67 ± 0.12) × 10^5[^ ^b^ ^]^	2.04 ± 0.1 × 10^5^
**AcO**	(1.89 ± 0.17) × 10^7[^ ^b^ ^]^	(1.66 ± 0.27) × 10^6[^ ^b^ ^]^	1.81 ± 0.2 × 10^6^
**AzureA**	(6.24 ± 0.53) × 10^7[^ ^e^ ^]^	(2.58 ± 0.17) × 10^6[^ ^a^ ^]^	5.19 ± 1.1 × 10^6^
**MB**	(1.24 ± 0.14) × 10^8[^ ^e^ ^]^	(1.16 ± 0.11) × 10^7[^ ^a^ ^]^	2.41 ± 0.4 × 10^7^
**MViolet**	(2.49 ± 0.25) × 10^5[^ ^a^ ^]^	(6.19 ± 0.54) × 10^4[^ ^a^ ^]^	5.76 ± 0.6 × 10^4^
**NeuRed**	(1.95 ± 0.24) × 10^6[^ ^a^ ^]^	(2.41 ± 0.11) × 10^5[^ ^a^ ^]^	2.62 ± 0.1 × 10^5^
**BCB**	(8.27 ± 0.15) × 10^5[^ ^a^ ^]^	(6.58 ± 0.16) × 10^5[^ ^a^ ^]^	–
**ThT**	(1.15 ± 0.04) × 10^6[^ ^b^ ^]^	(5.64 ± 0.08) × 10^4[^ ^b^ ^]^	1.39 ± 0.2 × 10^5^
**NDI+**	(1.82 ± 0.40) × 10^7[^ ^d^ ^]^	(1.10 ± 0.16) × 10^6[^ ^a^ ^]^	2.19 ± 0.2 × 10^6^
**PDI+**	(2.06 ± 0.13) × 10^6[^ ^a^ ^]^	(1.90 ± 0.06) × 10^5[^ ^a^ ^]^	1.81 ± 0.2 × 10^6^
**MV**	(2.59 ± 0.14) × 10^4[^ ^c^ ^]^	(1.12 ± 0.02) × 10^5[^ ^c^ ^]^	2.81 ± 0.2 × 10^5^

^[a]^ Direct UV/Vis titration of dye with host.

^[b]^ Direct fluorescence titration of dye with host.

^[c]^ Direct fluorescence titration of host with dye.

^[d]^ Competitive UV/Vis titration with **MViolet** as the competitor.

^[e]^ Competitive fluorescence titration with **ThT** as competitor. – not measured.

For some dyes (e.g., acridine orange (**AcO**), **PF**, and thioflavin T (**ThT**)), we used direct fluorescence titrations of dye with host. This choice was made because these dyes have insufficient UV/Vis intensity at the low µM concentrations required to avoid dye aggregation, whereas fluorescence spectroscopy displayed sufficient signal intensity at these concentrations. For example, Figure S30 shows the direct fluorescence titration of a fixed concentration of **ThT** (2.0 µM) with **H2** (0 – 10 µM), which shows a dramatic increase in **ThT** fluorescence intensity at 491 nm upon formation of the **H2**•**ThT** complex. This increase in fluorescence intensity can be attributed to the well‐known suppression of the conversion of the highly fluorescent locally excited state of **ThT** to the far less fluorescent twisted internal charge transfer (TICT) state upon conformational rigidification when present in more confined environments (e.g., as **H2**•**ThT**).^[^ ^16^ ^]^ Analogous direct fluorescence titrations were performed to measure the K_a_ values for **H2**•**ProF**, **H2**•**AcO**, **H1**•**ThT**, **H1**•**ProF**, and **H1**•**AcO** (Supporting Information Figures S9, S10, S16, S23, and S24).

**Figure 7:**
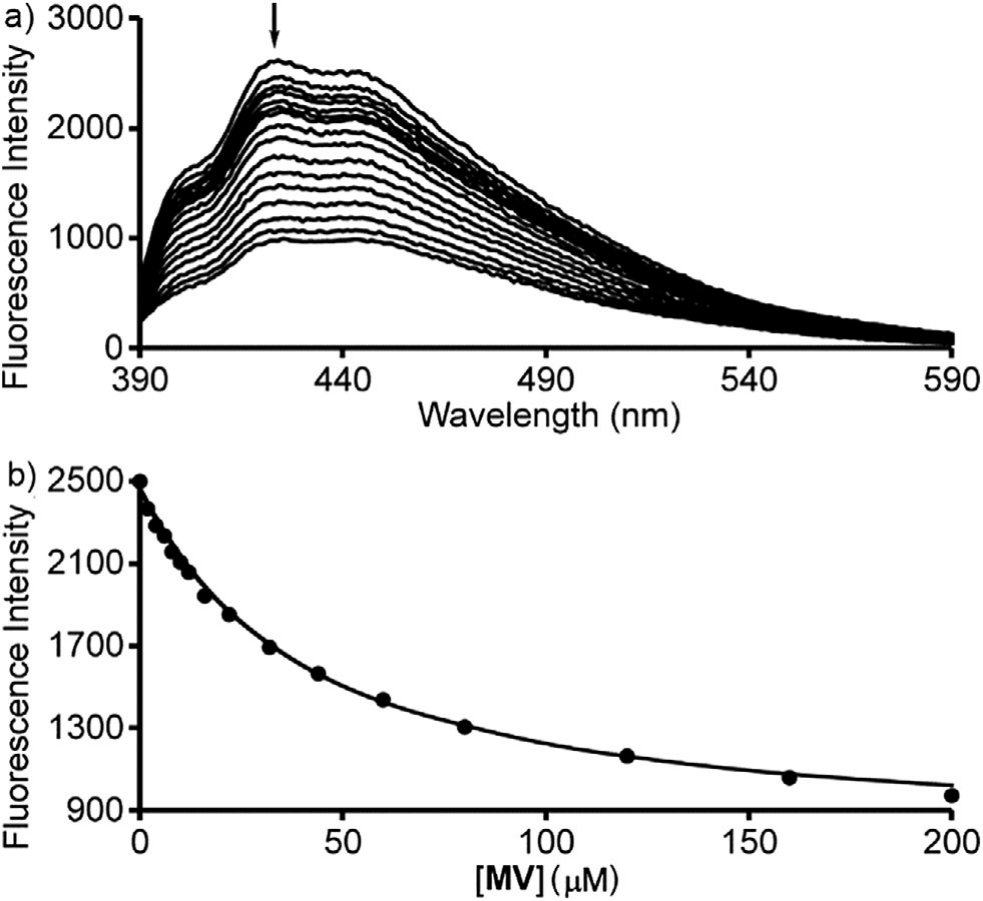
a) Plot of fluorescence intensity versus wavelength from the titration of **H2** (4 µM) with **MV** (0 – 200 µM). Excitation at 369 nm. b) Plot of fluorescence intensity at 435 nm versus [**MV**]. The solid line represents the best fit of the data to a 1:1 binding model implemented in Scientist^TM^ with K_a_ = 2.59 ± 0.14 × 10^4^ M^−1^.

For other host•dye complexes, the intricacies of the optical spectra dictated that we perform direct fluorescence titrations of fixed concentrations of hosts **H1** and **H2** with dye. For example, for methyl viologen (**MV**) and N‐methyl quinolinium (**NMeQ**), there is no wavelength where the dye can be independently observed by UV/Vis spectroscopy or independently excited for fluorescence spectroscopy. Figure 7a shows the fluorescence spectra (*λ*_ex_ = 369 nm) recorded during the titration of a fixed concentration of **H2** (4 µM) with methyl viologen (**MV**, 0 – 200 µM). The formation of the **H2**•**MV** complex results in quenching of the anthracene fluorescence. Figure 7b shows a plot of fluorescence intensity at 435 nm versus [**MV**]. The solid line represents the best nonlinear least squares fit of the data to a 1:1 binding model with K_a_ = 2.59 ± 0.14 × 10^4^ M^−1^ (Table 1). Analogous direct fluorescence titrations of host with dye were performed for **H2**•**NMeQ** and **H1**•**MV** (Figures S19, S20) and the K_a_ values are given in Table 1.

**Figure 8:**
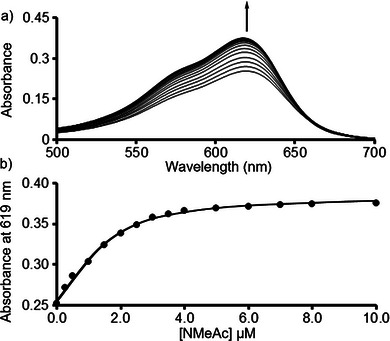
a) UV/Vis spectra recorded during the competitive titration of a solution of **MViolet** (3 µM) and **H2** (2.0 µM) with **NMeAc** (0 – 10 µM). b) Plot of A_619_ versus [**NMeAc**]. The solid line represents the best fit of the data to a competitive binding model implemented in Scientist^TM^ with K_a_ = (1.07 ± 0.10) x 10^7^ M^−1^.

For the **H2**•acridine (**Ac**), **H2**•**NMeAc**, **H2**•**NDI+**, and **H2**•**NMeQ** complexes neither direct UV/Vis titration nor direct fluorescence titration was appropriate because there is no wavelength at which **H2** or **Ac**, **NmeAc**, **NDI+**, or **NMeQ** could be selectively observed or excited. As shown in Figure 8a, we turned to a competitive UV/Vis titration of a solution of **H2** (2.0 µM) and **MViolet** (20 µM) in the cuvette with a solution of dye and **H2** (2.0 µM). We selected **MViolet** as the competitor for this experiment because of its moderate binding affinity (Table 1) and the strong UV/Vis signal generated upon displacement from **H2**. Figure 8b shows a plot of absorbance at 619 nm versus [**NMeAc**]. The solid line represents the best nonlinear least squares fit of the data to a competitive binding model using the previously determined values of K_a_ for **H2**•**MViolet** and the total concentrations of **H2** and **MViolet** as known inputs. In this manner, we determined the **H2**•**NMeAc** as 1.07 ± 0.10 × 10^7^ M^−1^ (Table 1).

Finally, in the case of **H2**•**MB** and **H2**•**AzureA,** we performed competitive fluorescence titrations using **ThT** as the competitor. This selection was dictated by the fact that the **H2**•**MB** and **H2**•**AzureA** complexes are too tight to be measured by direct titrations and because **ThT** can be selectively excited at wavelengths in between those of the anthracene‐walled host and the long wavelength blue absorbances of **MB** and **AzureA**. These competitive fluorescence titrations are given in the Supporting Information (Figures S25, S26), which allowed us to determine the K_a_ values for the **H2**•**MB** and **H2**•**AzureA** complexes as (1.24 ± 0.14) x 10^8^ and (6.24 ± 0.53) x 10^7^ M^−1^ respectively (Table 1).

### 2.6 Discussion of Trends in Binding Affinity

An examination of Table 1 shows that **H1** and **H2** bind to the panel of dyes with a range of *K*_a_ values (**H1**: 6340 M^−1^ – 1.16 × 10^7^ M^−1^; **H2**: 1.50 × 10^4^ M^−1^ – 1.24 × 10^8^ M^−1^), which enables a discussion of some factors governing the molecular recognition preferences of **H2**. In this section, we discuss the influence of: 1) the π‐extension from naphthalene‐walled (**H1**) to anthracene‐walled (**H2**) host on binding affinity, 2) π‐extension of the guest on K_a_, 3) intracavity electrostatic (e.g., ion‐quadrupole) effects, 4) the charge and nature of groups at the ureidyl carbonyl portals, and 5) the influence of the ionic strength of the phosphate buffer.

#### 2.6.1 Influence of π‐Extension of Aromatic Walls of Host on Host•Dye Binding Affinity

The amount of π‐surface area of the aromatic walls increases as one proceeds from **H1** to **H2**. Experimentally, Table 1 shows that **H2** frequently, but not always, binds stronger than **H1** to the members of the dye panel; the differences in K_a_ are as follows: **NMeQ**: 2.4‐fold, **Ac**: 63.3‐fold, **NMeAc**: 41.5‐fold, **ProF**: 10.4‐fold, **AcO**: 11.4‐fold, **AzureA**: 24.2‐fold, **MB**: 10.7‐fold, **MViolet**: 4.0‐fold, **NeuRed**: 8.1‐fold, **BCB**: 1.3‐fold, **ThT**: 20.4‐fold, **NDI+**: 16.54‐fold, **PDI+**: 10.8‐fold, **MV**: 0.23‐fold. It is well known that the hydrophobic driving force for complexation events is related to the amount of hydrophobic surface area that is buried. For example, Honig estimated the surface area dependence of the hydrophobic effect as ‐47 cal mol^−1^ Å^−2^, whereas Tanford suggested a value of ‐20 to ‐25 cal mol^−1^ Å^−2^.^[^ ^17^ ^]^ Most relevant to our work is Whitesides’ report that the ΔΔG of ligand binding due to benzo‐extension amounts to ‐20 cal mol^−1^ K^−1^.^[^ ^18^ ^]^ We calculate the increase in π‐surface area from **H1** to **H2** as 46.5 Å^2^ per wall based on MMFF minimized models of naphthalene and anthracene, which have surface areas of 161.5 and 208.0 Å^2^, respectively. Simple arithmetic – using the crude approximation that half of the additional π‐surface area is buried in the complex – and the values from Honig, Tanford, or Whitesides, respectively, suggest that **H2** should bind dyes approximately 2.19 kcal mol^−1^, 1.163, or 0.93 kcal mol^−1^ stronger than **H1**. These ΔG values correspond to 40‐fold, 7.1‐fold, or 4.8‐fold differences in binding affinity at room temperature. The observed differences in binding affinity given above are bracketed by calculated differences given above.

#### 2.6.2 Influence of π‐Extension of Guest on Host•Guest Binding Affinity

It is also possible to examine the influence of π‐extension of the dye on binding affinity toward a specific host. Within the dataset presented here, only **NMeQ and NMeAc** can be cleanly compared. Table 1 shows that **H2** binds 717‐fold stronger to **NMeAc** than to **NMeQ**. In contrast, **H1** binds 41‐fold stronger to **NMeAc** than to **NMeQ**. These experimental differences in binding affinity due to guest π‐extension are larger than that observed for the analogous host π‐extension described. We suspect that the larger differences in binding constant may be due to the fact that **NMeAc** binding is more substantially driven by ion‐quadrupole interactions with host. Guest **PDI **+ can be considered as a π‐extended version of **NDI +** but examination of Table 1 shows that both **H2** and **H1** bind the smaller **NDI + **approximately 10‐fold weaker than **PDI + **. Unfortunately, **NDI +** and **PDI +** differ in two structural variables (π‐surface area and distance between cationic N‐atoms), which precludes further analysis.

#### 2.6.3 Influence of Intracavity Cation‐π Effects on Host•Guest Binding Affinity

**H1** and **H2** are U‐shaped hosts with a roughly parallel orientation of their dialkoxyaromatic sidewalls and are therefore poised to engage in cation‐π interactions with planar aromatic guests that are cationic. The importance of cation‐π interactions on chemical and biological recognition processes has been established by Dougherty and co‐workers^[^ ^19^ ^]^ and used by Klärner and co‐workers to explain the strong binding of NAD + toward their benzonorbornene‐derived molecular clips.^[^ ^20^ ^]^ Within our dataset, neutral **Ac** and its cationic analogue **NMeAc** can be compared to shed light on the importance of cation‐π effects. Table 1 shows that **H2** binds **NMeAc** 20‐fold stronger than **Ac**. Similarly, **H1** binds **NMeAc** 31‐fold stronger than **Ac**. Another comparison can be made between cationic **MB** and a neutral analogue **MViolet**. Table 1 shows that **H2** binds 497‐fold stronger to **MB** than to **MViolet** and that **H1** displays a 187‐fold preference for **MB**. These larger differences in binding affinity for the **MB** / **MViolet** pair can be ascribed to cation‐π interactions and unfavorable interactions of the C = O group of **MViolet** and the ureidyl carbonyl portals of **H1** and **H2**.

(1) $$A_{\mathrm{obs}}=\frac{A_{\mathrm{AH}+}^{\infty }}{\left(1+10^{\mathrm{pH}-\mathrm{pKa}}\right)}+\frac{A_{A}^{\infty }}{\left(1+10^{\mathrm{pKa}-\mathrm{pH}}\right)}$$

**Figure 9:**
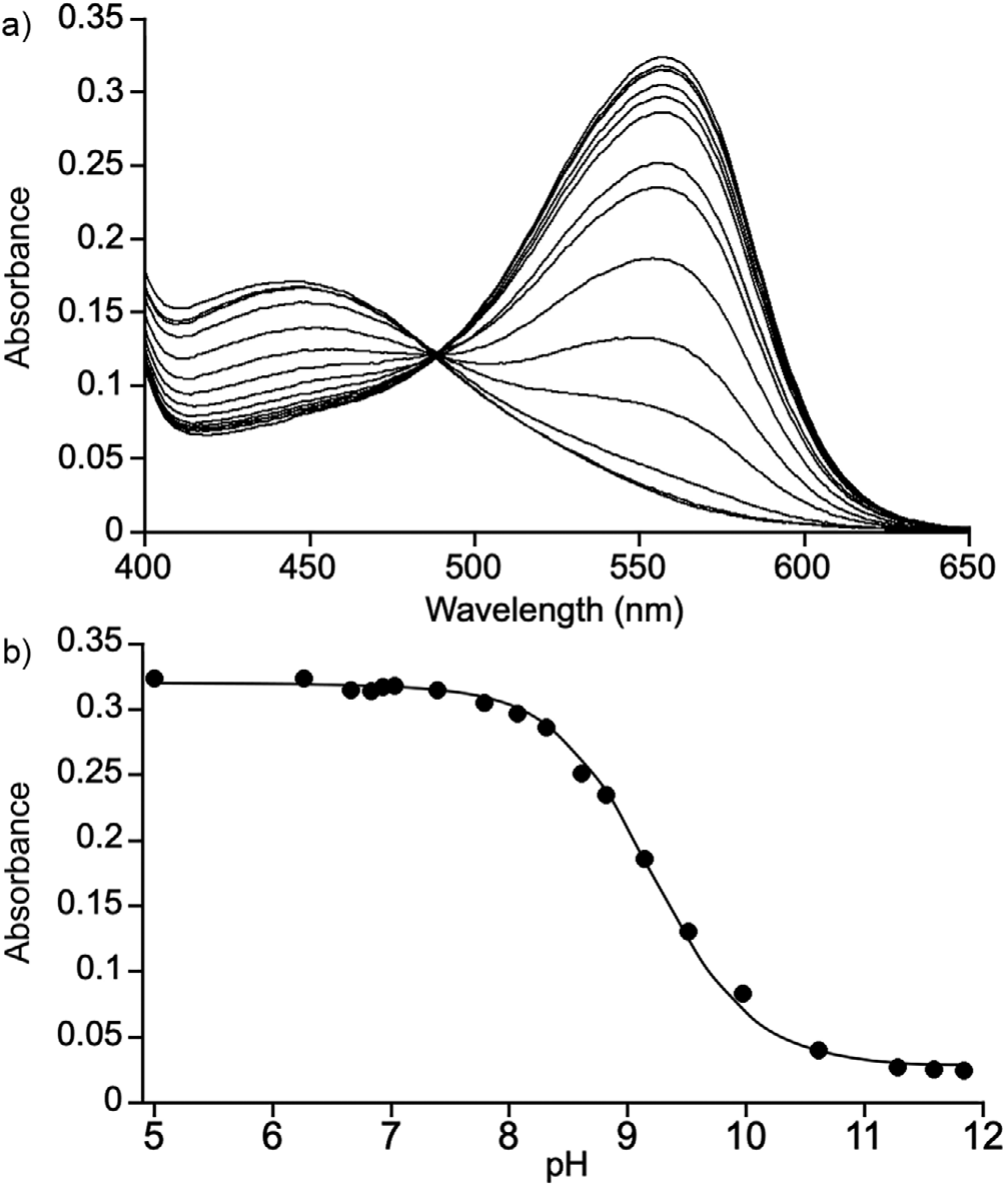
a) UV/Vis spectra recorded (room temperature, H_2_O) during the pH titration of a solution of **NeuRed** (20 µM) and **H2** (20 µM), b) a plot of absorbance at 557 nm versus pH fitted to eq. 1.

A separate assessment of the importance of cation‐π interactions on the molecular recognition of **H2** is possible using **NeuRed** as dye. **NeuRed** is used as a pH indicator that is red at pH 6.8 and becomes yellow at pH 8.0.^[^ ^21^ ^]^ When we performed a direct UV/Vis titration of **NeuRed** with **H2** at pH 7.4 in PBS, we observed the opposite color change from yellow to red, which strongly suggested that the pK_a_ of **NeuRed**•H^+^ is shifted within the **H2**•**NeuRed**•H^+^ complex. The p*K*_a_ of **NeuRed**•H^+^ was previously determined by pH titration to be 6.8.^[^ ^22^ ^]^ To determine the magnitude of this pK_a_ shift we measured UV/Vis spectra of an equimolar mixture of **H2** and **NeuRed** in water as a function of pH from pH 5 to pH 11 in triplicate (Figure 9a).^[^ ^23^ ^]^ The absorbance versus pH data (Figure 9b) can be fitted to equation 1 to determine the pK_a_ of the **H2**•**NeuRed**•H^+^ complex as 9.16 (average of triplicates). Based on a standard thermodynamic cycle argument the change in pK_a_ between **NeuRed**•H^+^ and **H2**•**NeuRed**•H^+^ is equal to the difference in log K_a_ for **H2**•**NeuRed** and **H2**•**NeuRed**•H^+^ (ΔpK_a_ = 2.36). Using this method, we calculate that **NeuRed**•H^+^ binds 229‐fold stronger than **NeuRed** to **H2**. This 3.21 kcal mol^−1^ difference in binding free energy can be attributed in large part to the cation‐π interaction in **H2**•**NeuRed**•H^+^. Previously, the **H1**•**NeuRed**•H^+^ complex was determined to be 48‐fold stronger than the **H1**•**NeuRed** complex by this method.^[^ ^12^ ^]^ On the basis of all these comparisons, we conclude that the cation‐π interaction is an important driving force in the molecular recognition properties of **H1** and **H2**.

#### 2.6.4 Influence of the Charge and Nature of Groups at the Ureidyl Carbonyl Portals

It is well known that CB[n]•guest complexes benefit from ion‐dipole interactions between the ammonium ion and the ureidyl C = O dipole of CB[n].^[^ ^1b^ ^]^ Studies from Nau and co‐workers based on pK_a_ shifts show that cationic ammonium ion guests commonly bind 10^3^‐fold stronger than their neutral amine form, although enhancements up to 10^5^‐fold have been observed.^[^ ^24^ ^]^ Dyes **ProF** and **AcO** differ only in the nature of the amino substituent (NH_2_ vs. NMe_2_) which resides at the ureidyl C = O portal in their complexes with **H1** and **H2**. Interestingly, we find that the **H2**•**AcO** and **H1**•**AcO** complexes are 6.8‐fold and 6.2‐fold stronger than the **H2**•**ProF** and **H1**•**ProF** complexes, respectively. Similarly, the difference between **AzureA** and **MB** is the change from an NH_2_ to an NMe_2_ substituent. We find that the **H2**•**MB** and **H1**•**MB** complexes are 2.0‐fold and 4.5‐fold stronger than the **H2**•**AzureA** and **H1**•**AzureA** complexes, respectively. The known pK_a_ values of **AzureA** (p*K*_a_ = 12)^[^ ^25^ ^]^ and **MB** (p*K*_a_ of **MB**•H^+^ ≈ 0.5)^[^ ^26^ ^]^ establish that they are monocationic at pH 7.4. Therefore, the differences in K_a_ for the complexes of **H1** and **H2** with **MB** and **AzureA** are unlikely to be due to cation‐π interactions. We believe that the more diffuse nature of the charged NMe_2_ groups allows it to form stronger electrostatic interactions with the ureidyl C = O portals via N^+^CH_3_•••O = C interactions; a related effect has been documented in the recognition behavior of CB[7].^[^ ^27^ ^]^ Alternatively, the lipophilic nature of the NMe_2_ relative to the NH_2_ group may also play a role in the observed stronger binding.

#### 2.6.5 Influence of the Ionic Strength of the Phosphate Buffer

The influence of buffer identity and ionic strength often plays a significant role in molecular recognition events in water.^[^ ^28^ ^]^ Within the group of macrocyclic CB[n] receptors it is well known that the presence of high concentrations of alkali metal salts reduces the CB[n]•guest binding affinity due to competitive binding of metal ion at the ureidyl carbonyl portals of the host.^[^ ^29^ ^]^ For example, Nau and co‐workers showed that CB[6] binds sixfold weaker to cyclohexylmethyl ammonium ion in 0.2 M Na_2_SO_4_ than in D_2_O.^[^ ^30^ ^]^ Accordingly, we have added to Table 1 the K_a_ values for various **H1**•dye complexes measured previously in 20 mM sodium phosphate buffer.^[^ ^12^ ^]^ Overall, Table 1 shows that the binding constants measured in 20 mM sodium phosphate buffered water are slightly stronger (two–threefold) or up to 10‐fold stronger (**NDI+**) than those measured in PBS. Given that PBS contains high concentrations of NaCl (137 mM), we attribute these changes to competitive binding of Na^+^ at the ureidyl C = O portals of uncomplexed host.

## 3 Conclusion

In summary, we report the synthesis and characterization of a new anthracene‐walled molecular clip (**H2**). **H2** is nicely soluble in water (≥7 mM) and is monomeric at concentrations (< 500 µM) that are suitable for examination by optical spectroscopy. We also studied the binding of **H2** toward selected dyes by ^1^H NMR spectroscopy. Analysis of the complexation‐induced changes in chemical shift indicates that **MB**, **ProF**, and **NDI **+ bind inside the cleft of **H2** with the cationic groups residing near the ureidyl C = O groups of **H2**. For **NMeAc**, the NMe group is located inside the cavity with the geometry shown in Figure 3. We used direct or competitive UV/Vis or fluorescence titrations to determine the binding affinity of the panel of dyes toward **H2** and **H1** as comparators in biologically relevant PBS buffer (Table 1). In favorable cases (e.g., **H2**•**MB**), binding affinity can exceed 10^8^ M^−1^. We find that π‐extension of the walls from **H1** to **H2** results in an increase in binding constant by approximately one order of magnitude. The superior binding of cationic **NMeAc** over neutral **Ac** and **NeuRed•H + **over neutral **NeuRed** (e.g., ΔpK_a_ = 2.36) establishes that cation‐π interactions are an important driving force for **H2**•dye complexation. Dyes that possess NH_2_ substituents (e.g., **ProF**) display weaker binding toward **H2** than the analogous dyes with NMe_2_ substituents (e.g., **AcO**). In conclusion, rational changes to the structure of methylene‐bridged glycoluril‐dimer‐derived hosts and their guests can be used to enhance binding affinity. We expect that **H2**•dye complexes might prove useful in the creation of sensing ensembles for aromatics. Furthermore, **MB** is currently used as a nuclear stain, and it is expected that **H2** could be used as a destaining agent in such applications.

## Supporting Information

The authors have cited additional references within the Supporting Information.^[^ ^31^ ^]^

## Conflict of Interest

L.I. is co‐founder and holds equity in Reversal Therapeutics (College Park, Maryland) and holds equity in Clear Scientific (Cambridge, Massachusetts).

## Supporting information



Supporting Information

## Data Availability

Electronic versions of the data that support this publication can be downloaded from the Digital Repository at the University of Maryland (https://drum.lib.umd.edu/home) via the following DOI: 10.13016/pdpr‐fvnj.
