# Supplementary material for: Aromatic Wall Extension of Glycoluril‐Derived Molecular Clips Enhances Binding of Planar Aromatic Dyes
Source: Chemistry. 2025 Aug 18;31(50):e02177. doi: 10.1002/chem.202502177 (PMC12415320; doi:10.1002/chem.202502177)
Supplement: Supplementary file 1 — Supporting Information [file CHEM-31-e02177-s001.pdf]

Aromatic Wall Extension of Glycoluril-Derived Molecular Clips Enhances Binding of Planar Aromatic Dyes

*By Collin J. Vincent, David King, Steven L. Murkli, Lyle Isaacs**

Department of Chemistry and Biochemistry, University of Maryland,

College Park, Maryland 20742, United States

Supporting Information

Corresponding author: Lyle Isaacs, LIsaacs@umd.edu

Table of Contents Pages

General experimental details……………………………………………… S3

Electronic supporting data availability …………………………………… S3

Synthetic procedure and characterization data…………………………... S4 – S7

Determination of inherent solubility of **H2** ……………………………… S8

^{1}H NMR spectra of **H2** at different concentrations in water …………… S9

K_a determination for **H1** by UV/Vis and fluorescence spectroscopy …… S10 – S23

K_a determination for **H2** by UV/Vis and fluorescence spectroscopy …… S24 – S37

Dilution Experiment for **H2** by UV/Vis spectroscopy …………………… S38

^{1}H NMR spectra recorded for selected **H2**•dye complexes ……………… S39 – S41

Determination of quantum yield for **H2** ………………………………… S42

Binding Models Used to Determine K_a values with Scientist™………… S43 – S44

References cited ………………………………………………………… S45

General Experimental Details

Starting materials and all dyes were purchased from commercial suppliers were used without further purification. N-methyl acridinium was prepared according to the literature procedure.[S1] NMR spectra were measured on spectrometers operating at 400 or 600 MHz for ^{1}H and 100 or 150 MHz for ^{13}C NMR spectra. Routine mass spectrometry was performed using a JEOL AccuTOF electrospray instrument (ESI). UV/Vis spectra were measured on a Varian Cary 100 UV spectrophotometer. Fluorescence was measured on a Hitachi F-4500 Fluorescence spectrophotometer. UV/Vis and fluorescence titrations were conducted in duplicate. The temperature was maintained at 25 ˚C for all titration experiments using Lauda or Neslab recirculating baths.

Electronic Supporting Data Availability

The electronic data that support this publication can be freely downloaded from the Digital Repository at the University of Maryland (https://drum.lib.umd.edu/home) via the following DOI: 10.13016/pdpr-fvnj.

Synthetic Procedure and Characterization Data

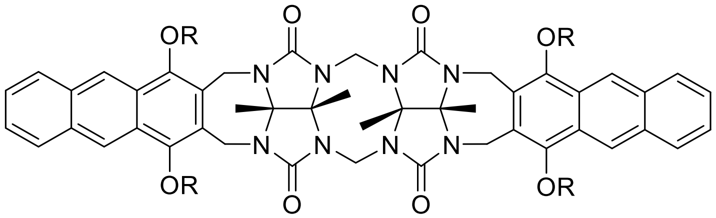

H2

H2: A mixture of **W1** (5.72 g, 11.6952 mmol) and **G2** (1.00 g, 2.339 mmol) was dissolved in TFA:Ac_2O (1:4, v:v; 50 mL) under N_2. The reaction mixture was stirred at 90 ˚C for 4 h. The reaction was quenched by pouring the crude reaction mixture into acetone (1 L, rapidly stirred) which gave a green precipitate instantly. The resulting suspension was stirred for 5 min. and then sonicated briefly to liberate all solids from the walls. The solids were isolated by multiple rounds of centrifugation and decanting (8000 rpm, 10 min, 50 mL centrifuge tubes (two at a time)). The resulting solid was washed three times with acetone (40 mL) by brief vortexing to dislodge the solid pellet and sonication for 5 min. during each washing cycle. The solid was dried on high vacuum overnight. The resulting solid was reprecipitated twice by dissolving in the minimum quantity of H_2O followed by pouring into acetone (20-fold volume of water used) which caused a precipitate to form immediately. The precipitate was obtained by iterative centrifugation (8000 rpm, 10 min., 2 x 50 mL tubes). The resulting solid was rinsed with acetone (40 mL) by vortexing to dislodge the pellet, sonicated for 5 min., and centrifuged (8000 rpm, 10 min). The supernatant was poured off to give crude solid. The crude solid was further purified by GPC (Sephedex G25, 100 g, water as eluent). The column fractions were monitored by TLC (CH_2Cl_2, 1% MeOH). The resulting solid was purified a second time using the same GPC column to remove dark green colored fractions eluting before the long-wave active UV fraction attributed to **H2**). The solid isolated (300 mg) from the second GPC column is slightly impure by NMR. The solid (300 mg) was dissolved in water (10 mL) and split into two 50 mL centrifuge tubes. The centrifuge tubes were swirled by hand and slowly filled to a total volume of 45 mL with CH_3CN. The solid was obtained by centrifugation (7500 rpm, 10 min) and the liquid was decanted. The solid was further washed with CH_3CN (30 mL per tube) by vortexing, sonicated for 5 min., and centrifuged (7500 rpm, 10 min.). The supernatant was poured off giving **H2** as a pale yellow-green solid after drying on high vacuum (0.210 g, 0.1737 mmol, 7.4%). M.p. > 300 ˚C. IR (ATR, cm^{-1}): 3431 br., 2946 w, 2882 w, 1713 m, 1465 m, 1428 m, 1392 w, 1341 m, 1309 m, 1179 s, 1076 m, 1029 s, 877 m, 842 m, 825 m, 788 m, 758 s, 709 m. ^1H NMR (400 MHz, DMSO-d_6): 8.42 (s, 4H), 8.01 (ABq, 4H), 7.44 (ABq, 4H), 5.60 (d, J = 15.8, 2H), 5.36 (d, J = 16.0, 4H), 4.35 (d, J = 16.0, 4H), 4.25-4.10 (m, 6H), 3.95-3.85 (m, 4H), 2.85-2.75 (m, 8H), 2.20-2.05 (m, 8H), 1.82 (s, 6H), 1.74 (s, 6H). ^{13}C NMR (100 MHz, D_2O): δ 155.9, 147.7, 131.5, 128.9, 127.2, 125.7, 125.1, 121.6, 78.3, 77.1, 73.4, 48.8, 48.2, 43.7, 36.2, 25.7, 16.2. ESI-MS: *m/z* 659.38 ([**H2** – 4Na + 2H]$^{2-}$, $C_{58}H_{62}N_8O_{20}S_4^{2-}$, calcd. 659.15); 681.34 ([**H2** – 2Na]$^{2-}$, $C_{58}H_{60}N_8O_{20}S_4Na_2^{2-}$, calcd. 681.13).

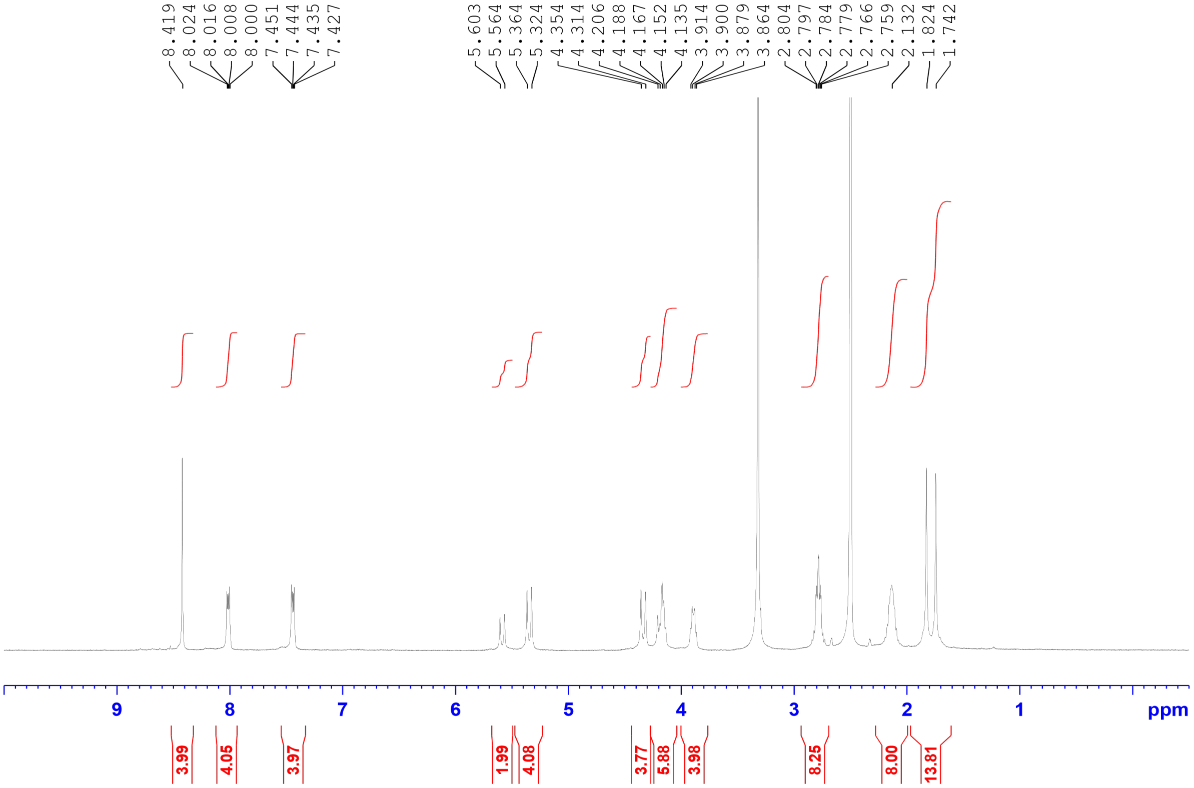

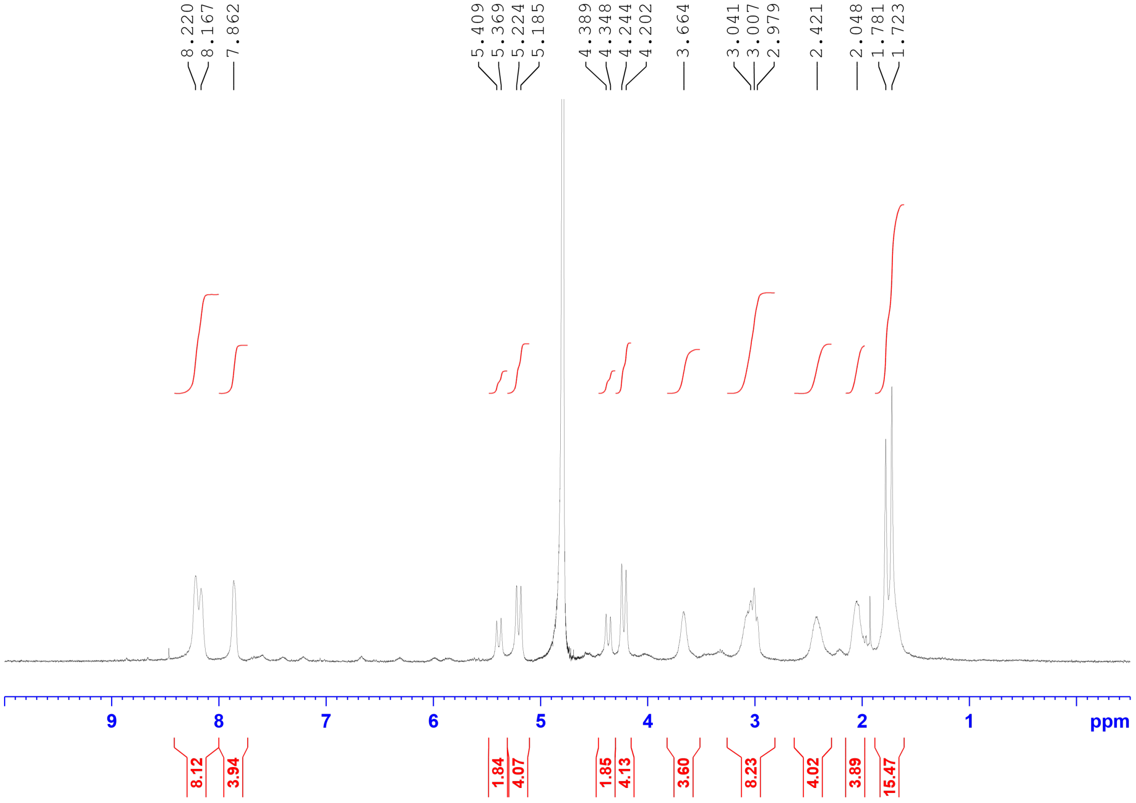

Figure S1. (Top) ^{1}H NMR spectra recorded (400 MHz, RT, DMSO-*d*$_6$) for **H2**. (Bottom) ^{1}H NMR spectra recorded (400 MHz, RT, D_2O) for **H2**.

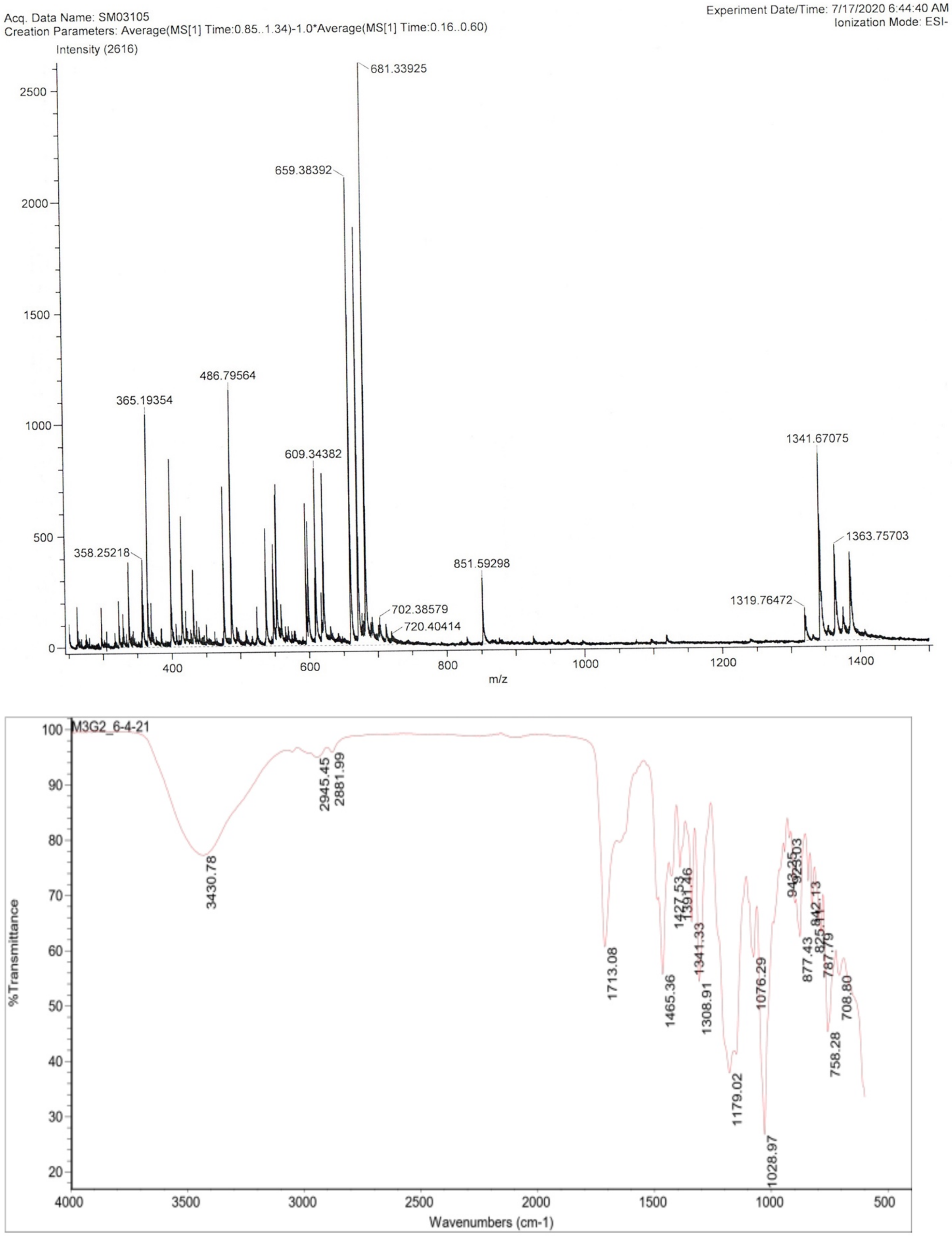

Figure S2. (Top) Mass spectrum (ESI, negative ion mode) recorded for **H2**. (Bottom) IR spectrum (ATR, cm^{-1}) recorded for **H2**.

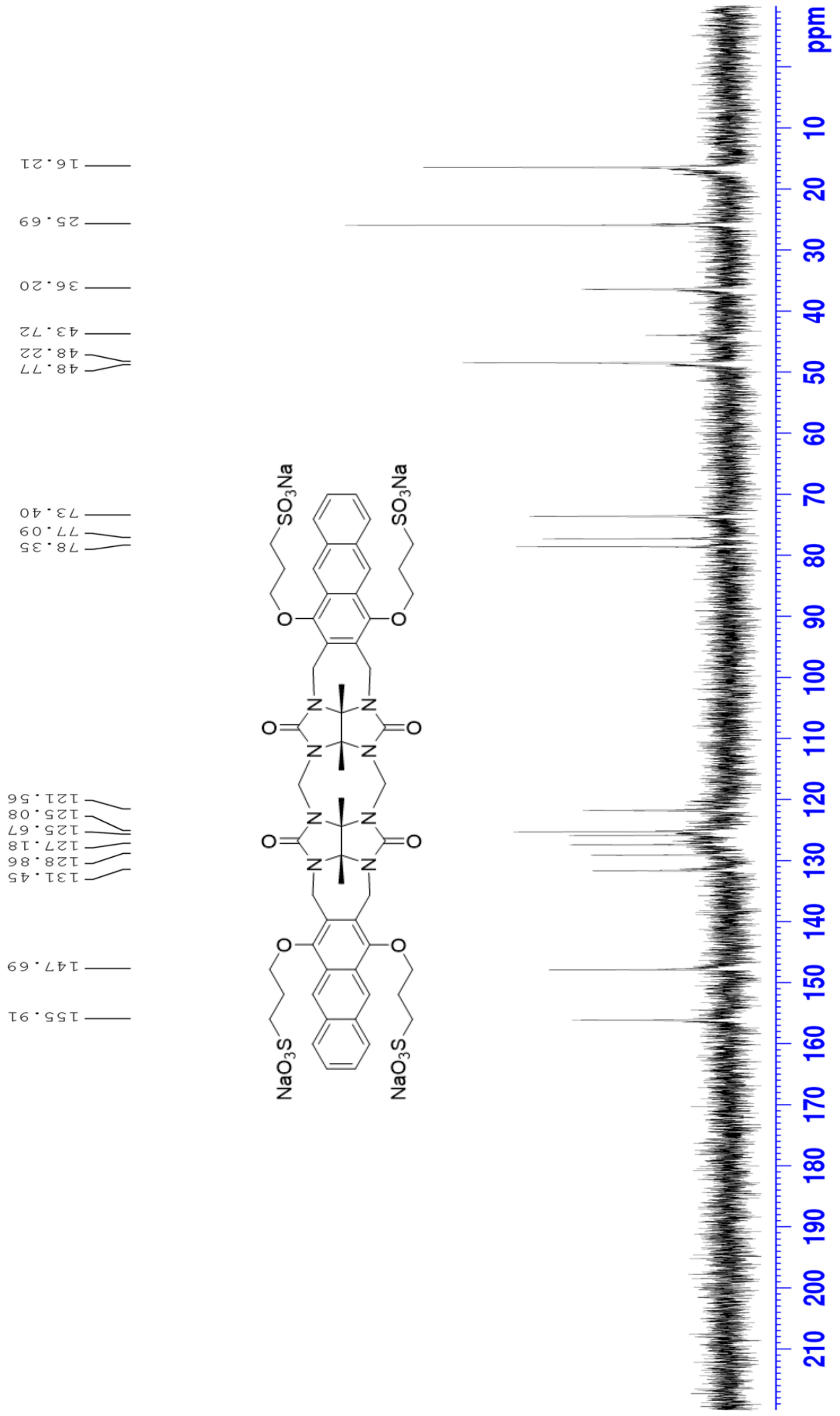

Figure S3. ^{13}C NMR spectra recorded (100 MHz, RT, D_2O) for **H2**.

Determination of inherent solubility of H2.

To an excess amount of **H2** was added 100 µL of deuterated water (D_2O). The mixture was vortexed and then sonicated for 5 minutes. The mixture was centrifuged, and the supernatant was collected. After a 50-fold dilution, dimethyl sulfone (0.5 mM) was added as an internal standard, and the ^1H NMR spectra of the solution was measured. The signal for the internal standard resonates at 3.117 ppm (s, 6H). Diagnostic signals for **H2** were integrated. The ratio of the integrals of the internal standard relative to the integrals of the host were used to determine the concentration of **H2** to be 6.995 mM.

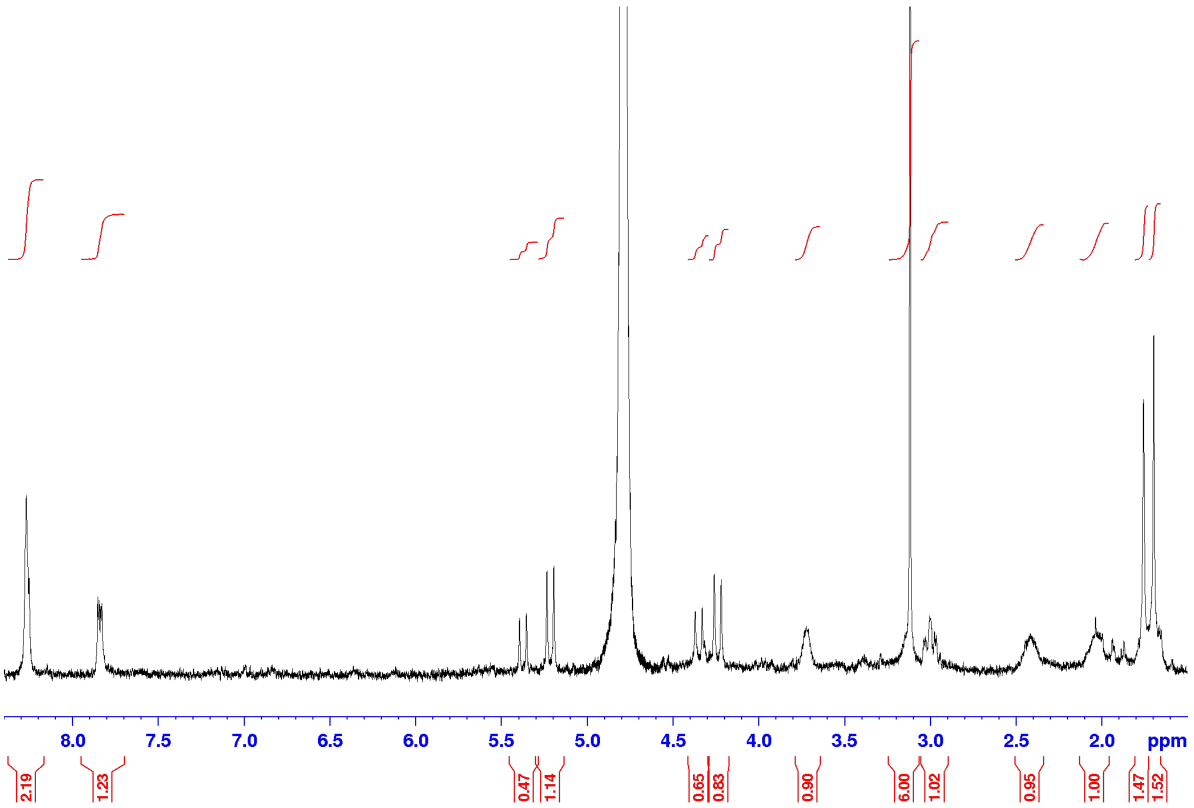

Figure S4. ^1H NMR spectra recorded (400 MHz, RT, D_2O) for **H2** and dimethyl sulfone (0.5 mM).

^{1}H NMR spectra of H2 at different concentrations in water.

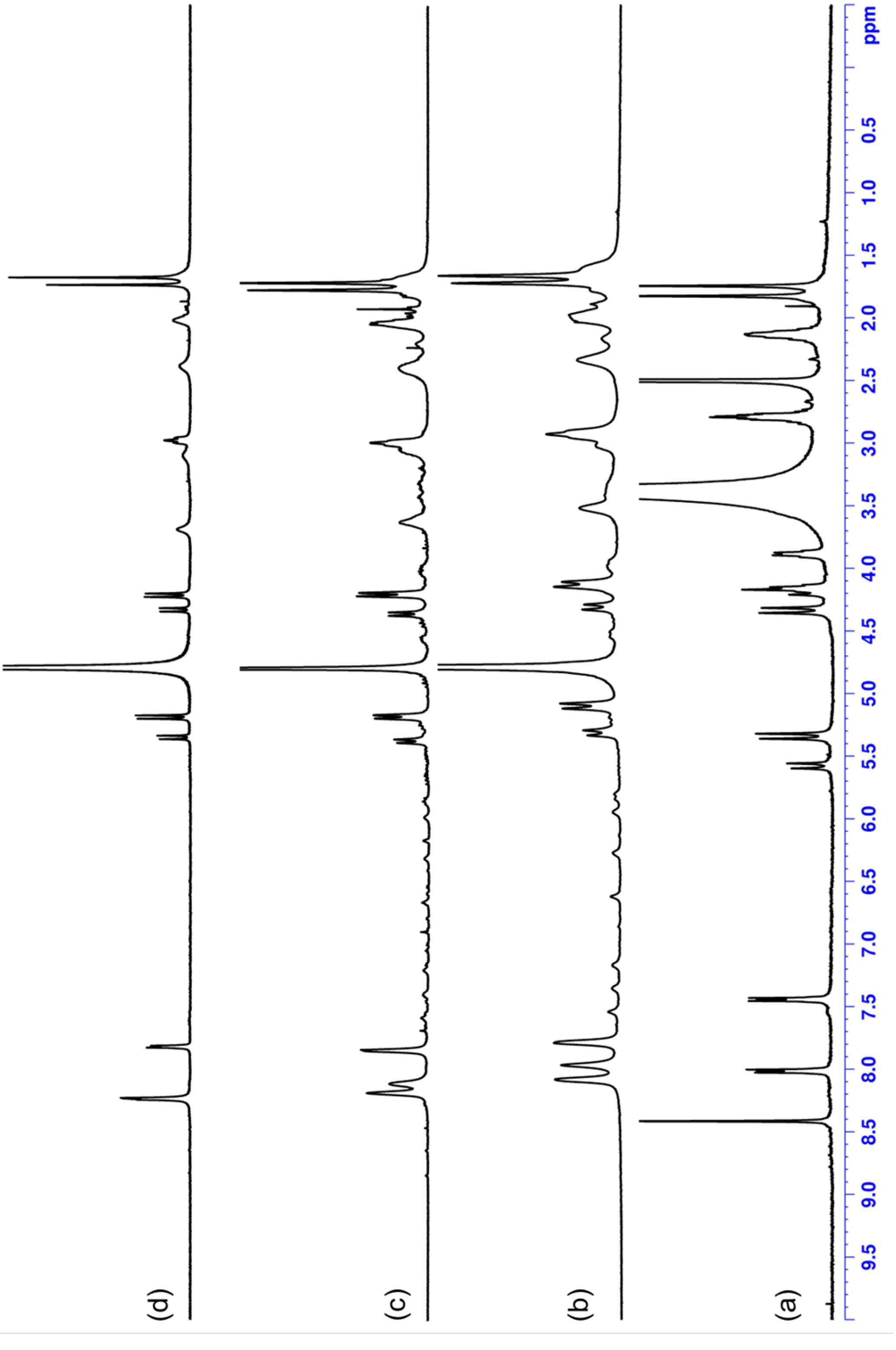

Figure S5. a) ^{1}H NMR spectra recorded (400 MHz, RT, DMSO-d_6) for **H2** at 2.8 mM. ^{1}H NMR spectra recorded (400 MHz, RT, D_2O) for **H2** at: b) 4.0 mM. c) 2.8 mM, d) 0.5 mM.

K_a determination for H1 by UV/Vis and Fluorescence spectroscopy

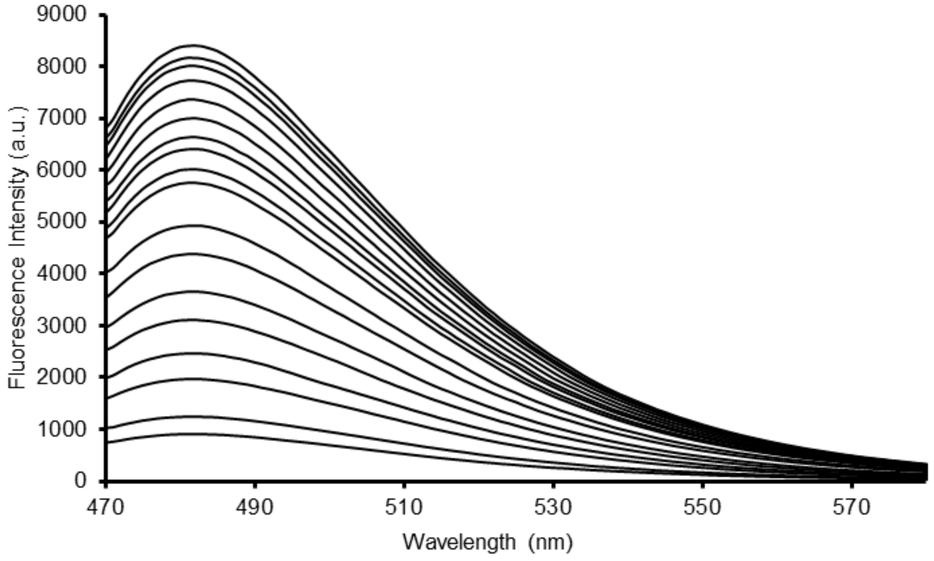

a)

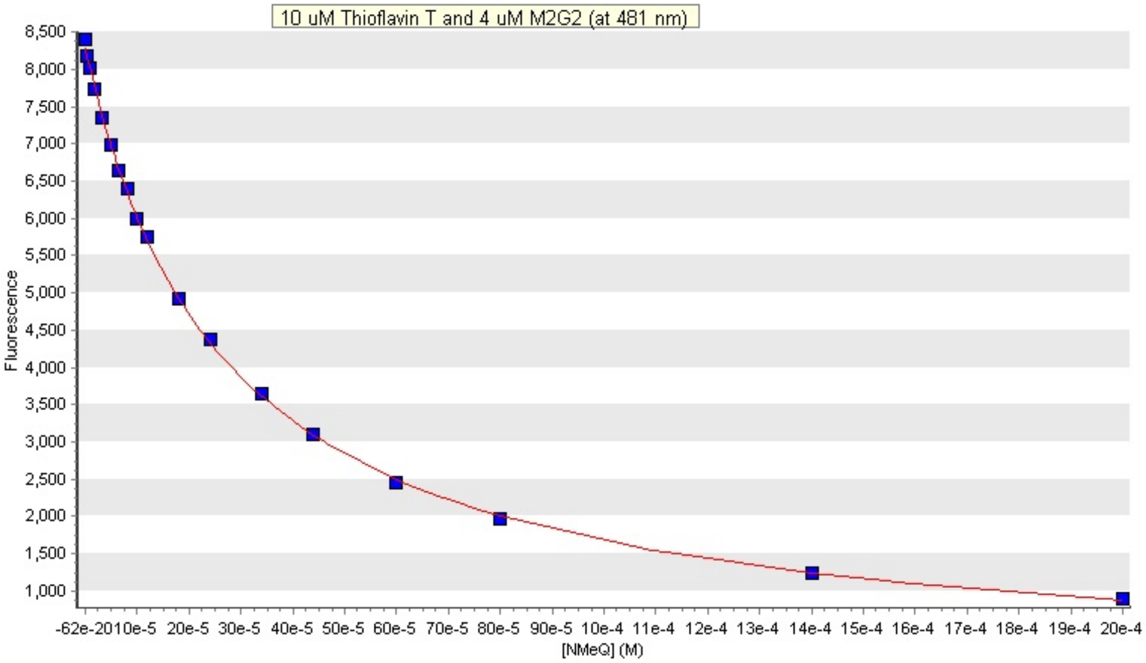

b)

Figure S6. a) Fluorescence spectra from the competitive titration of **ThioflavinT** (10.0 μM) and **H1** (4.0 μM) with **NMQ** (0-2000 μM) (Conditions: PBS buffer, pH 7.4, RT); b) Plot of emission intensity at 481 nm (ex. 444 nm) versus [**NMQ**]. The solid curve represents the best non-linear fit of the data to a competitive binding model implemented in Scientist™ with $K_a = (6.34 \pm 0.08) \times 10^3$ M^{-1}.

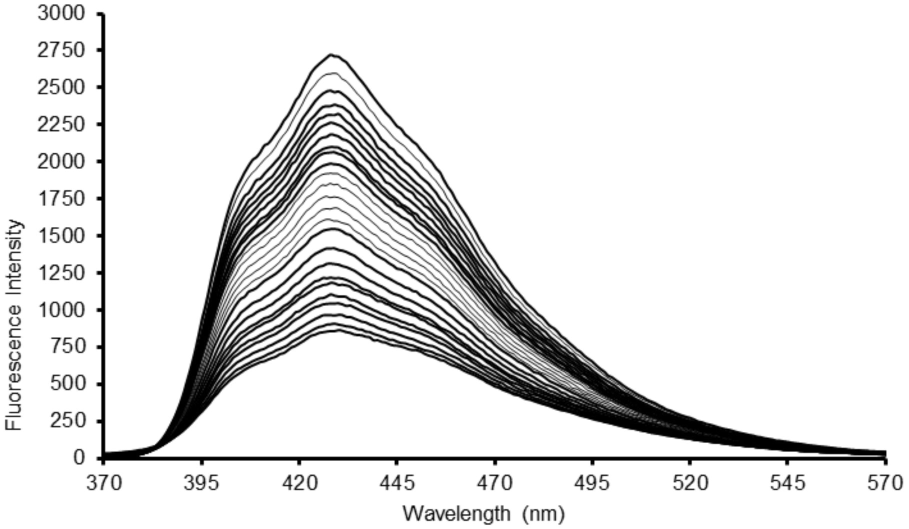

a)

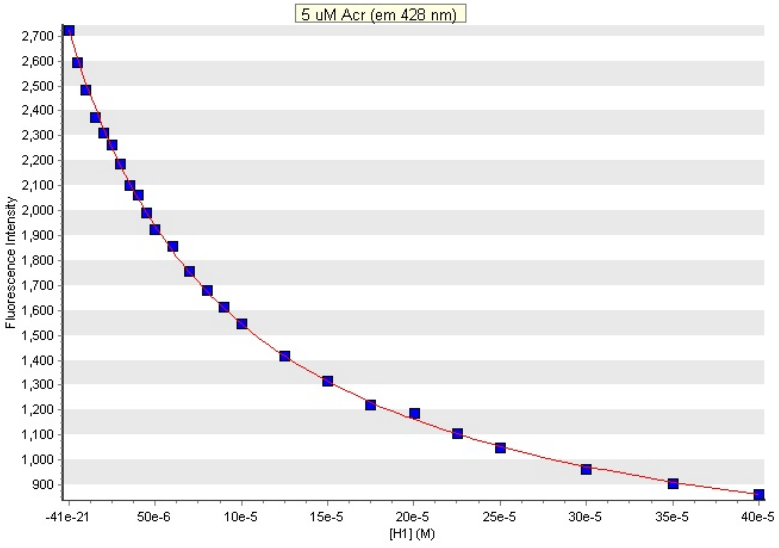

b)

Figure S7. a) Fluorescence spectra from the direct titration of **Ac** (5.0 μM) with **H1** (0-100 μM) (Conditions: PBS buffer, pH 7.4, RT); b) Plot of emission intensity at 428 nm (ex. 355 nm) versus [**H1**]. The solid curve represents the best non-linear fit of the data to a 1:1 binding model implemented in Scientist™ with $K_a = (8.31 \pm 0.12) \times 10^3$ M^{-1}.

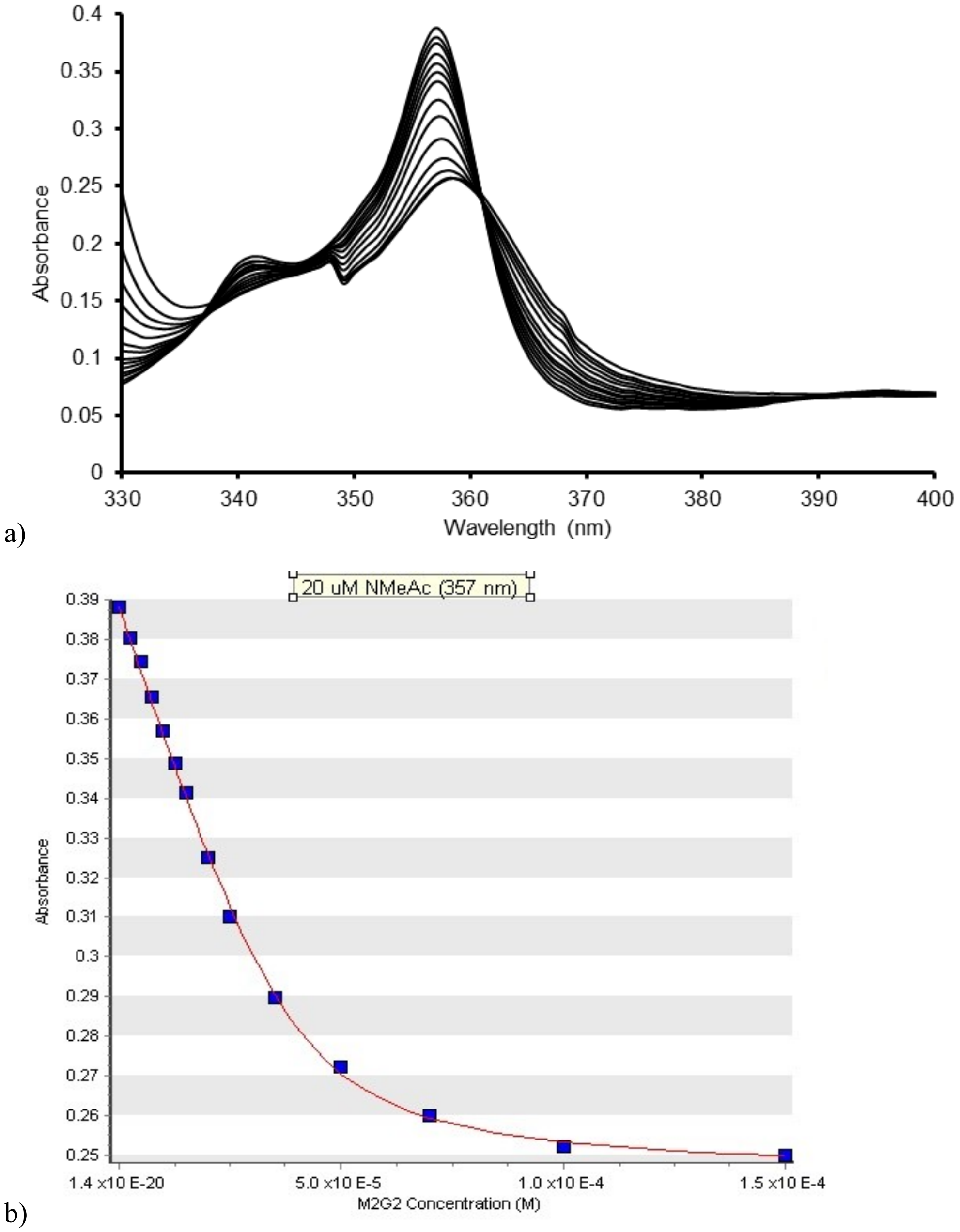

Figure S8. a) UV/Vis spectra from the direct titration of **NMeAc** (20.0 μM) and **H1** (0-150 μM) (Conditions: PBS buffer, pH 7.4, RT); b) Plot of absorbance at 357 nm versus [**H1**]. The solid curve represents the best non-linear fit of the data to a 1:1 binding model implemented in Scientist™ with $K_a = (2.59 \pm 0.36) \times 10^5$ M^{-1}.

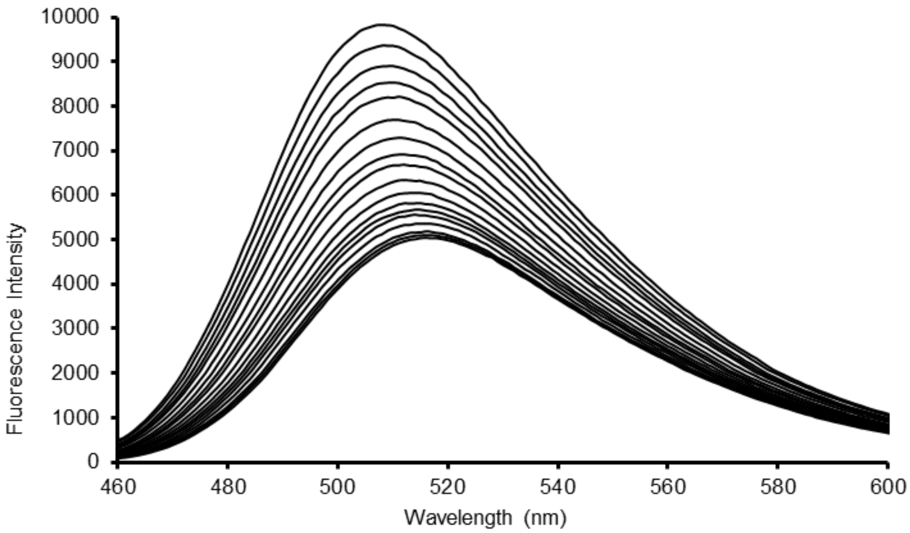

a)

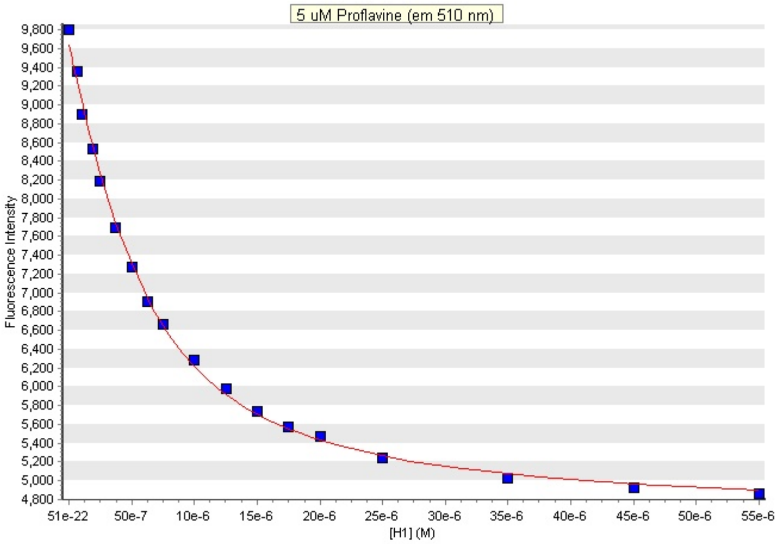

b)

Figure S9. a) Fluorescence spectra from the direct titration of **ProF** (5.0 μM) with **H1** (0-55.0 μM) (Conditions: PBS buffer, pH 7.4, RT); b) Plot of emission intensity at 510 nm (ex. 444 nm) versus [**H1**]. The solid curve represents the best non-linear fit of the data to a 1:1 binding model implemented in Scientist™ with $K_a = (2.67 \pm 0.12) \times 10^5$ M^{-1}.

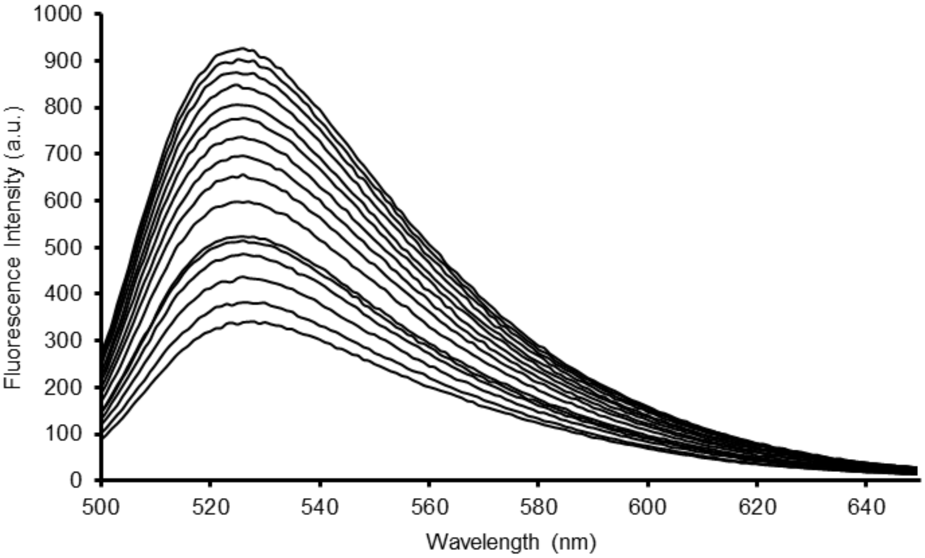

a) \

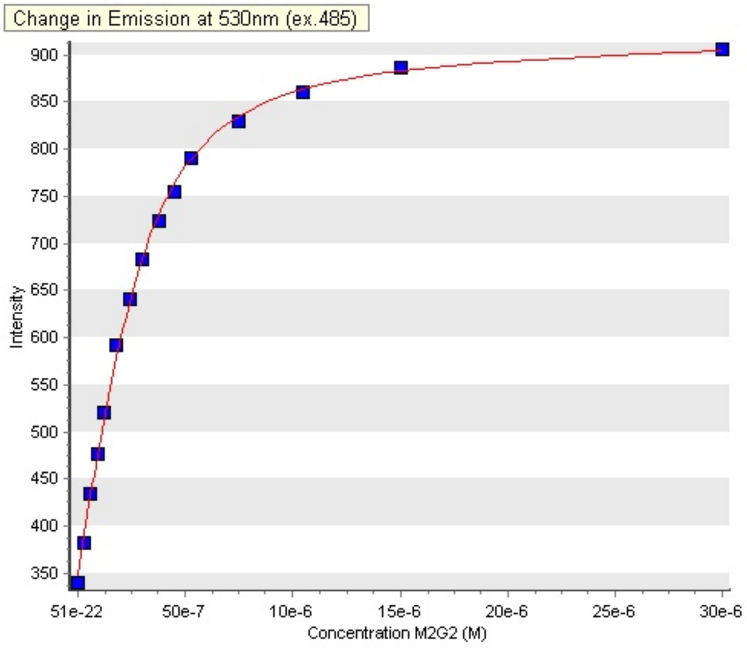

b)

Figure S10. a) Fluorescence spectra from the direct titration of **AcO** (3.0 μM) with **H1** (0-30.0 μM) (Conditions: PBS buffer, pH 7.4, RT); b) Plot of emission intensity at 530 nm (ex. 485 nm) versus [**H1**]. The solid curve represents the best non-linear fit of the data to a 1:1 binding model implemented in Scientist™ with $K_a = (1.66 \pm 0.27) \times 10^6\ M^{-1}$.

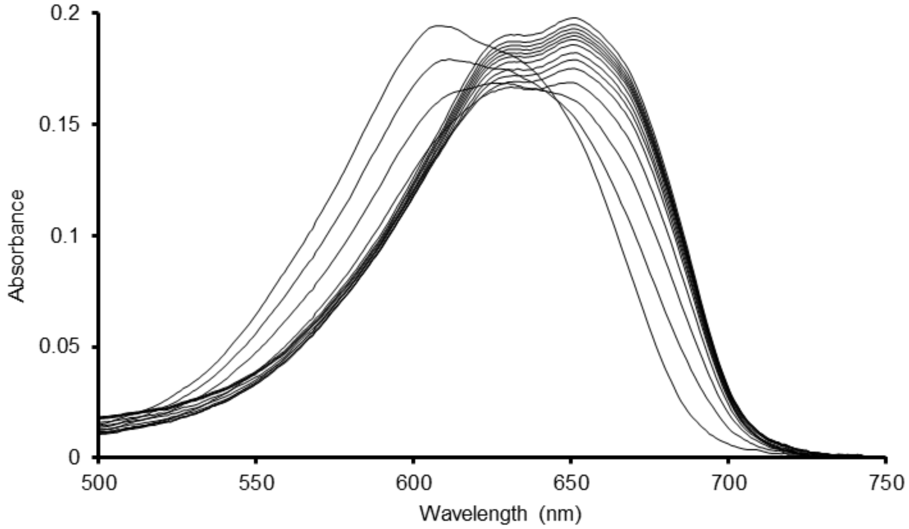

a)

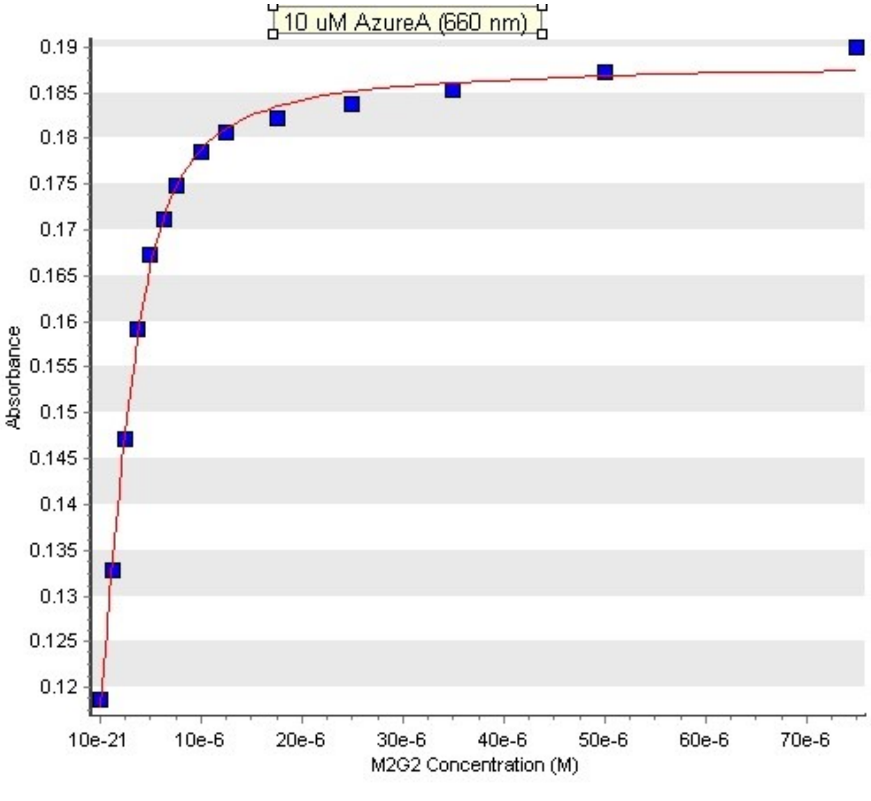

b)

Figure S11. a) UV/Vis spectra from the direct titration of **AzureA** (10.0 μM) with **H1** (0-75.0 μM) (Conditions: PBS buffer, pH 7.4, RT); b) Plot of absorbance intensity at 660 nm versus [**H1**]. The solid curve represents the best non-linear fit of the data to a 1:1 binding model implemented in Scientist^{TM} with $K_a = (2.58 \pm 0.17) \times 10^6$ M^{-1}.

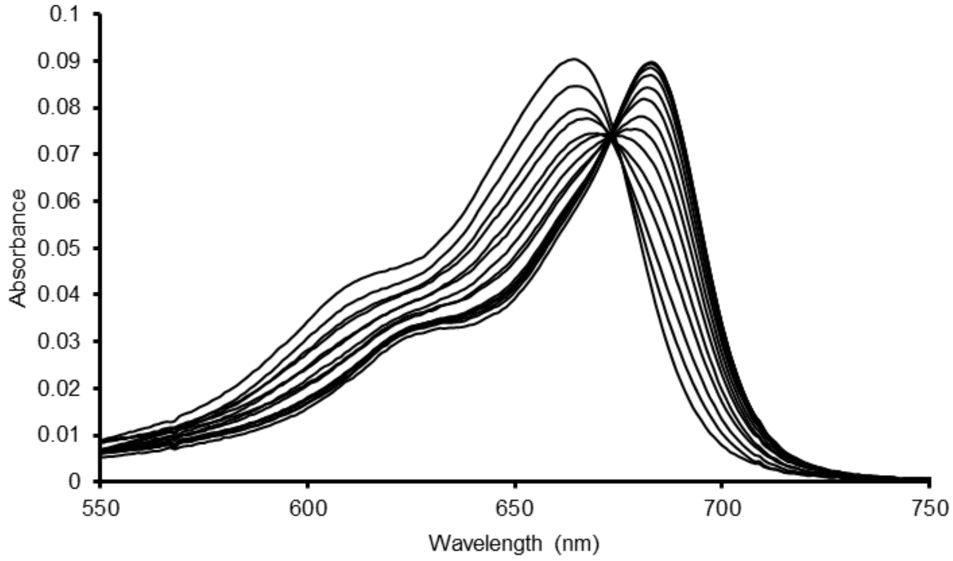

a)

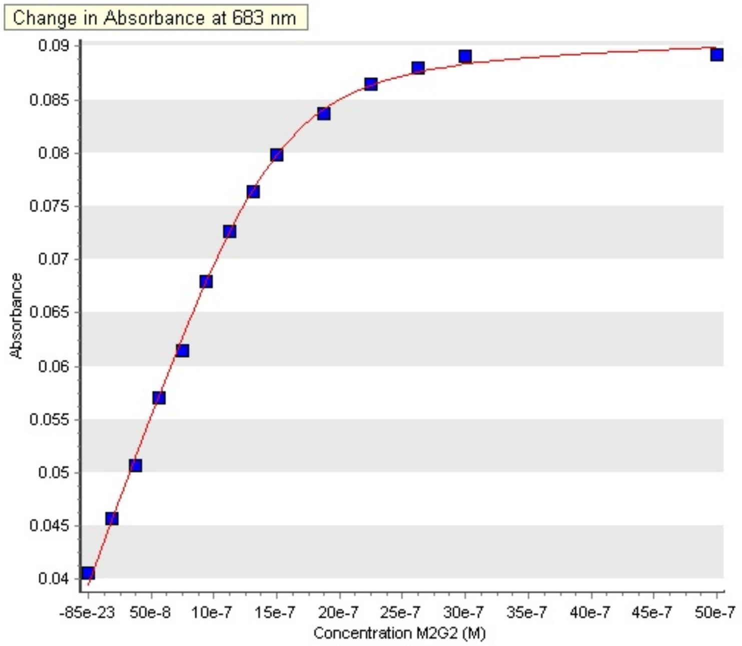

b)

Figure S12. a) UV/Vis spectra from the direct titration of **MB** (1.5 μM) with **H1** (0-5.0 μM) (Conditions: PBS buffer, pH 7.4, RT); b) Plot of absorbance at 683 nm versus [**H1**]. The solid curve represents the best non-linear fit of the data to a 1:1 binding model implemented in Scientist™ with $K_a = (1.16 \pm 0.11) \times 10^7$ M^{-1}.

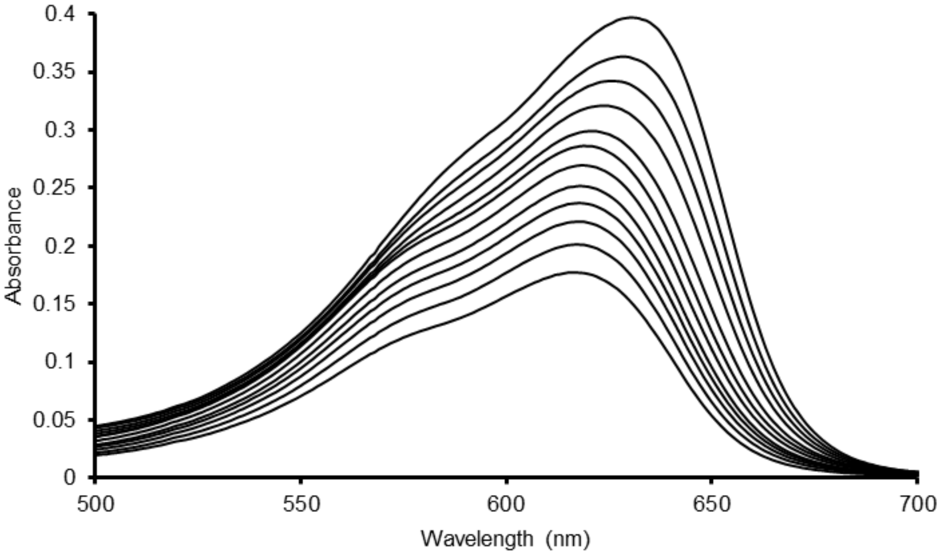

a)

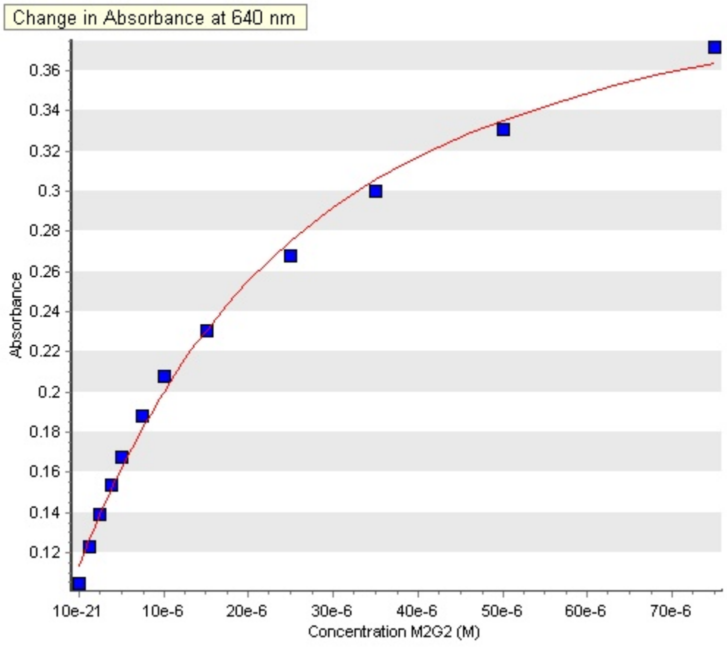

b)

Figure S13. a) UV/Vis spectra from the direct titration of **MViolet** (10.0 μM) with **H1** (0-75.0 μM) (Conditions: PBS buffer, pH 7.4, RT); b) Plot of absorbance at 640 nm versus [**H1**]. The solid curve represents the best non-linear fit of the data to a 1:1 binding model implemented in Scientist^{TM} with K_a = $(6.19 \pm 0.54) \times 10^4$ M^{-1}.

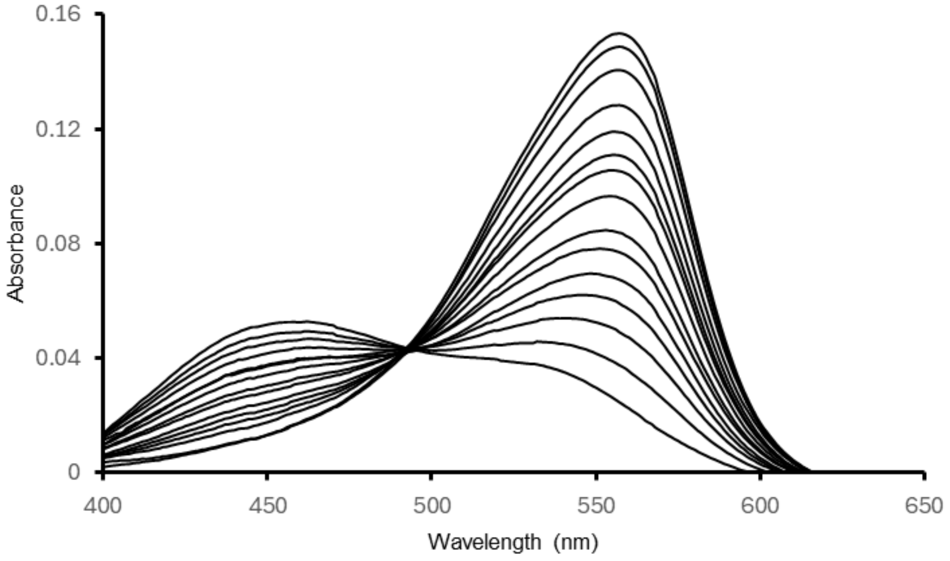

a)

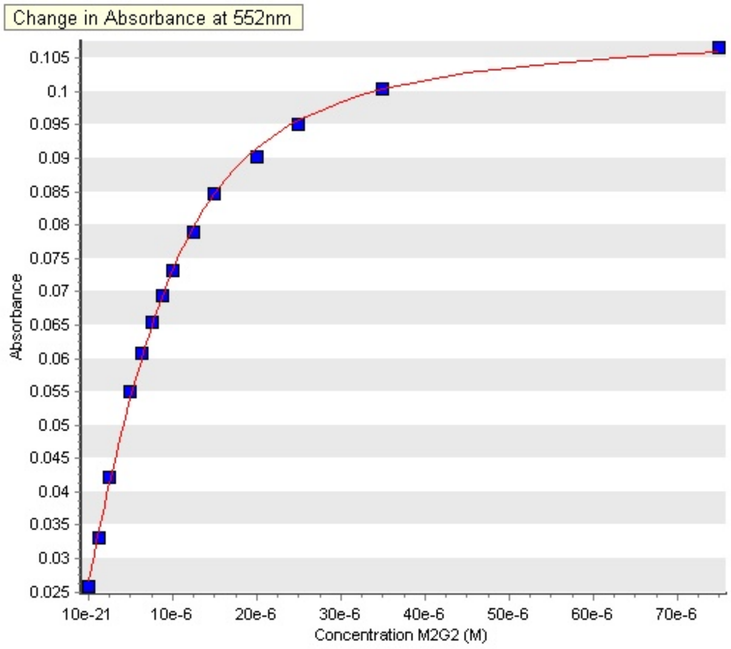

b)

Figure S14. a) UV/Vis spectra from the direct titration of **NeuRed** (10.0 μM) with **H1** (0-72.5 μM) (Conditions: PBS buffer, pH 7.4, RT); b) Plot of absorbance at 562 nm versus [**H1**]. The solid curve represents the best non-linear fit of the data to a 1:1 binding model implemented in Scientist^{TM} with K_a = (2.41 ± 0.11) × 10^5 M^{-1}.

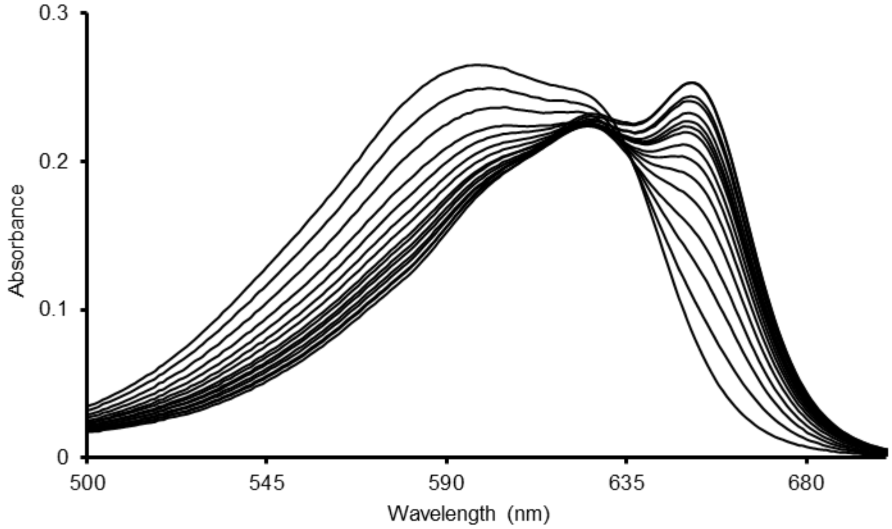

a)

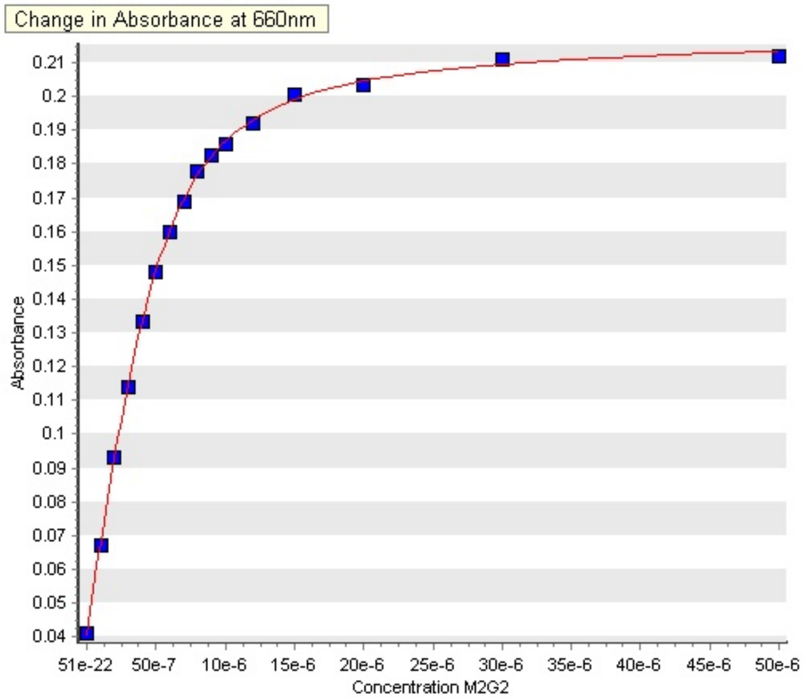

b)

Figure S15. a) UV/Vis spectra from the direct titration of **BCB** (5.0 µM) with **H1** (0-50.0 µM) (Conditions: PBS buffer, pH 7.4, RT); b) Plot of absorbance at 660 nm versus [**H1**]. The solid curve represents the best non-linear fit of the data to a 1:1 binding model implemented in Scientist™ with $K_a = (6.58 \pm 0.16) \times 10^5$ M^{-1}.

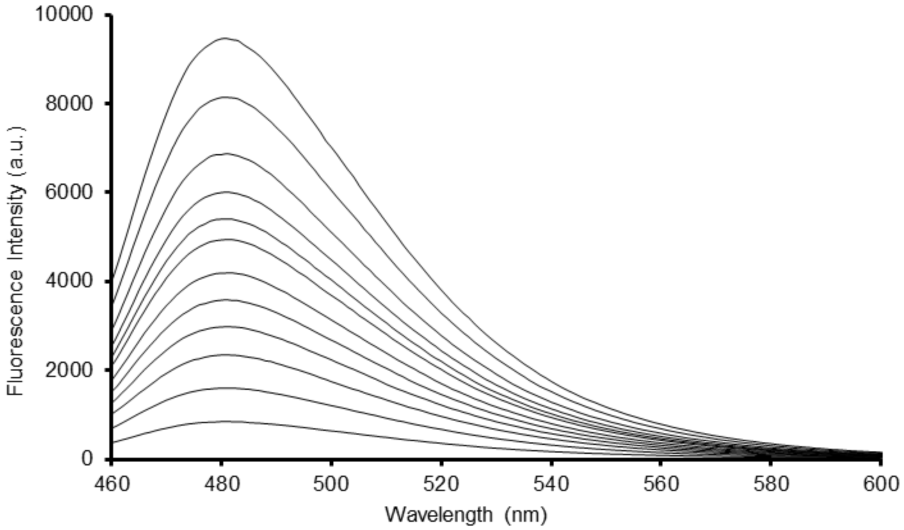

a)

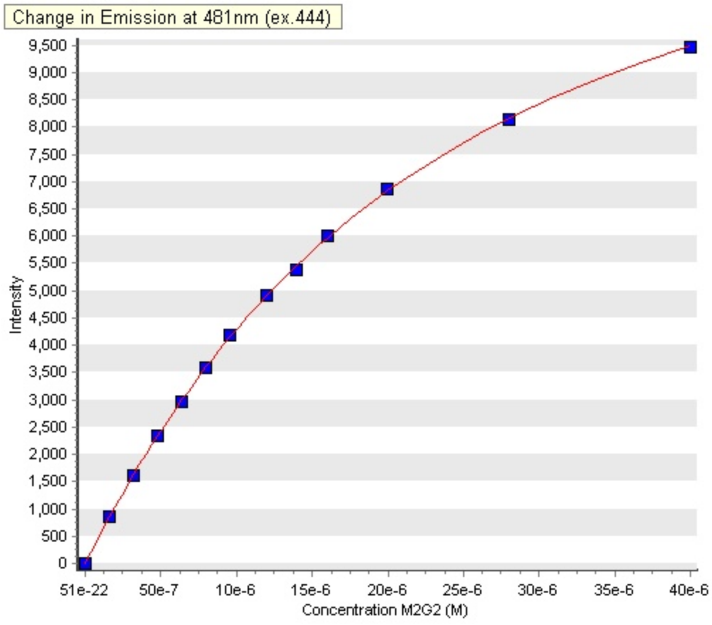

b)

Figure S16. a) Fluorescence spectra from the direct titration of **ThT** (8.0 μM) with **H1** (0-60.0 μM) (Conditions: PBS buffer, pH 7.4, RT); b) Plot of emission intensity at 481 nm (ex. 450 nm) versus [**H1**]. The solid curve represents the best least squares regression of a non-linear fit of the data to a 1:1 binding model implemented in Scientist^{TM} with $K_a = (5.64 \pm 0.08) \times 10^4$ M^{-1}.

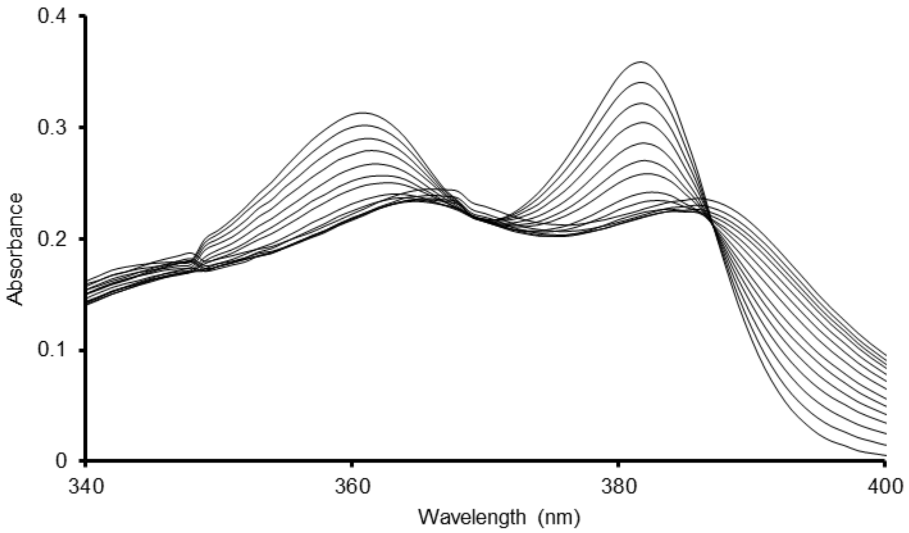

a)

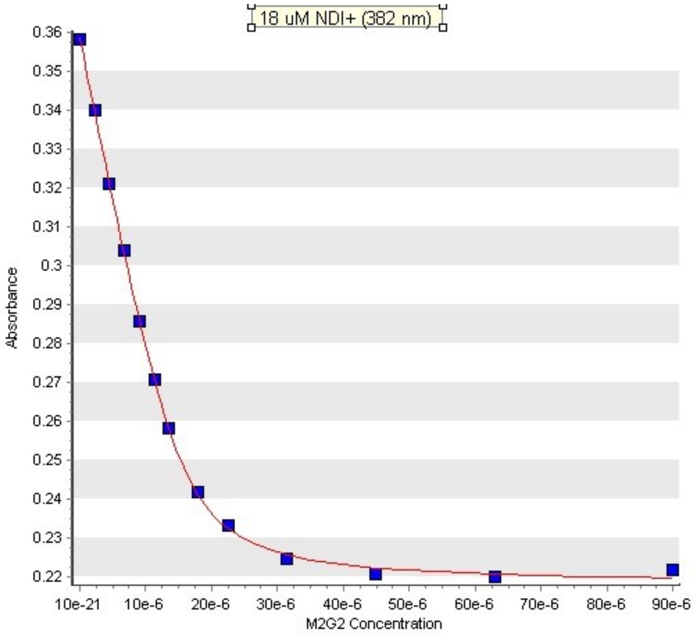

b)

Figure S17. a) UV/Vis spectra from the direct titration of **NDI+** (18.0 μM) with **H1** (0-90.0 μM) (Conditions: PBS buffer, pH 7.4, RT); b) Plot of absorbance at 382 nm versus [**H1**]. The solid curve represents the best least squares regression of a non-linear fit of the data to a 1:1 binding model implemented in Scientist™ with $K_a = (1.10 \pm 0.16) \times 10^6\ M^{-1}$.

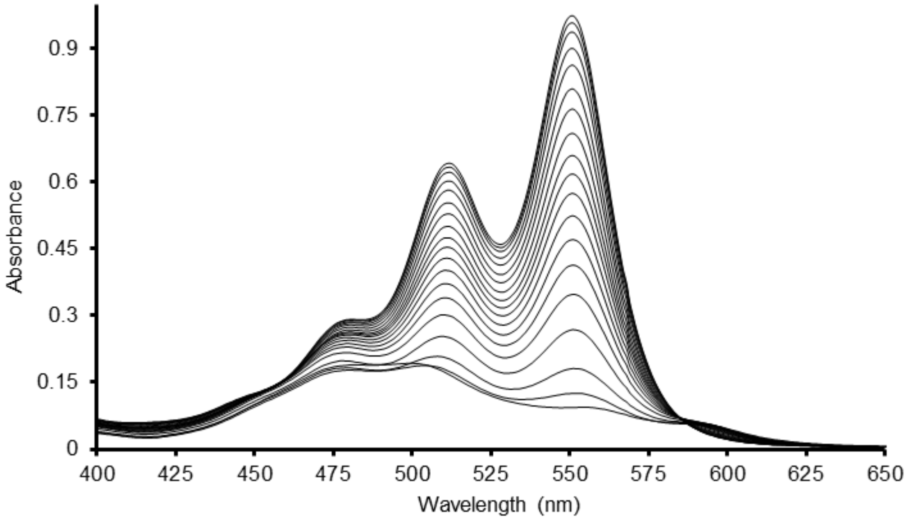

a)

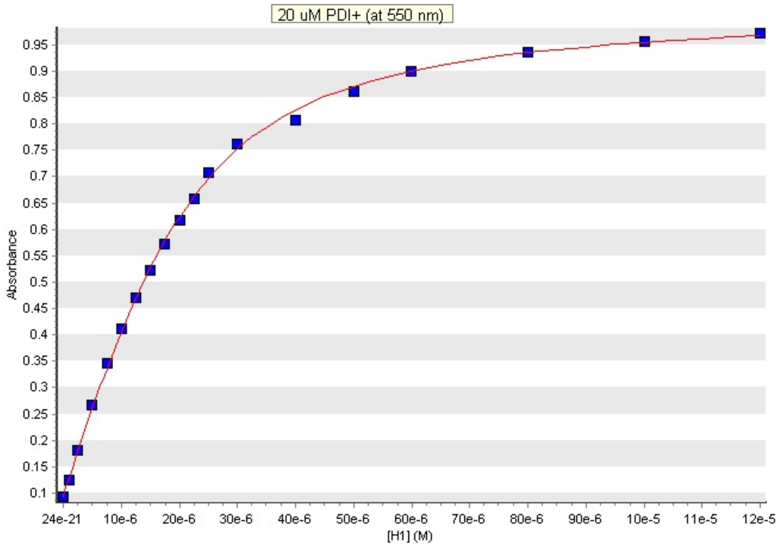

b)

Figure S18. a) UV/Vis spectra from the direct titration of **PDI+** (20 μM) with **H1** (0-120 uM) (Conditions: PBS buffer, pH 7.4, RT); b) Plot of absorbance at 550 versus [**H1**]. The solid curve represents the best non-linear fit of the data to a 1:1 binding model implemented in Scientist™. K_a was evaluated as $(1.90 \pm 0.06) \times 10^5\ M^{-1}$.

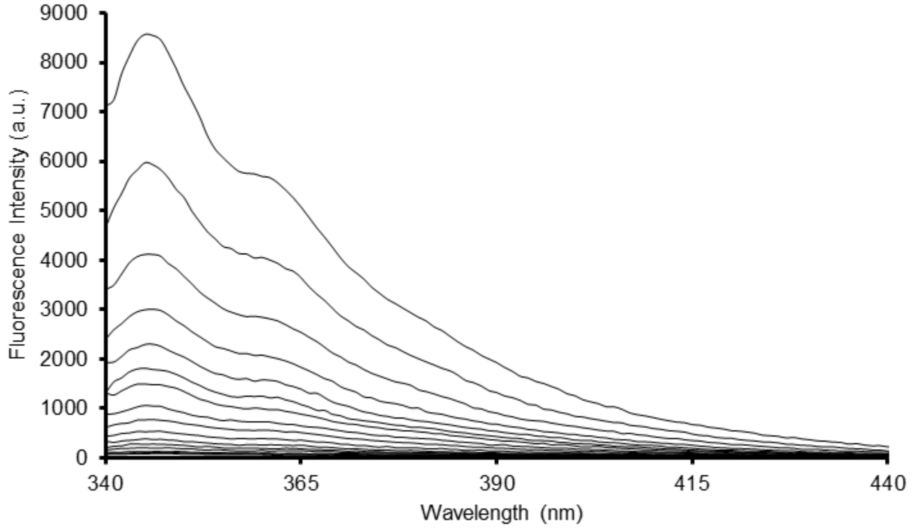

a)

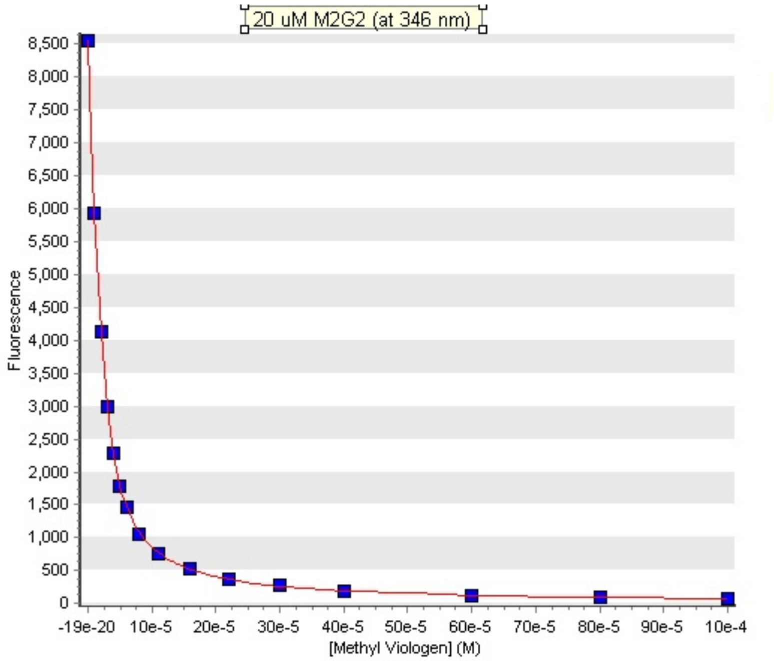

b)

Figure S19. a) Fluorescence spectra from the direct titration of **H1** (20.0 μM) with **Methyl Viologen** (0-1000.0 μM) (Conditions: PBS buffer, pH 7.4, RT); b) Plot of emission intensity at 346 nm (ex. 325 nm) versus [**MV**]. The solid curve represents the best least squares regression of a non-linear fit of the data to a 1:1 binding model implemented in Scientist™ with $K_a = (1.12 \pm 0.02) \times 10^5\ M^{-1}$.

K_a determination for H2 by UV/Vis and Fluorescence spectroscopy

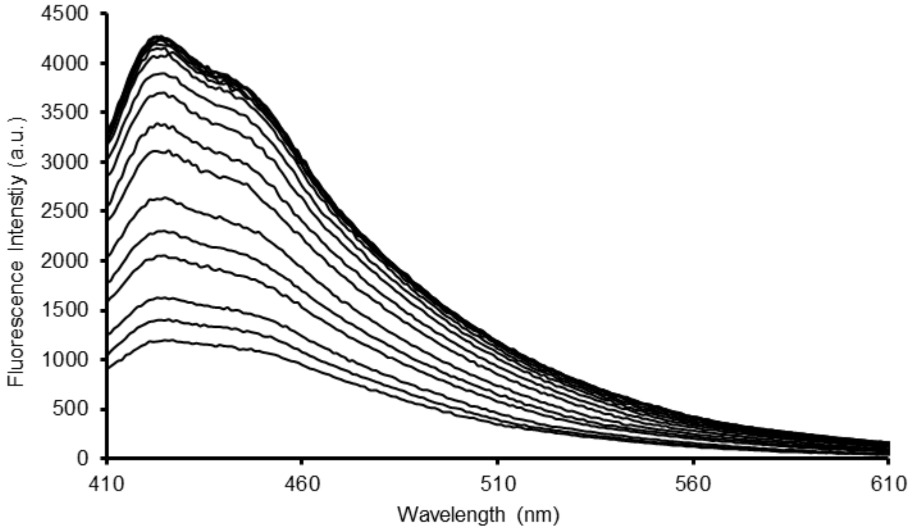

a)

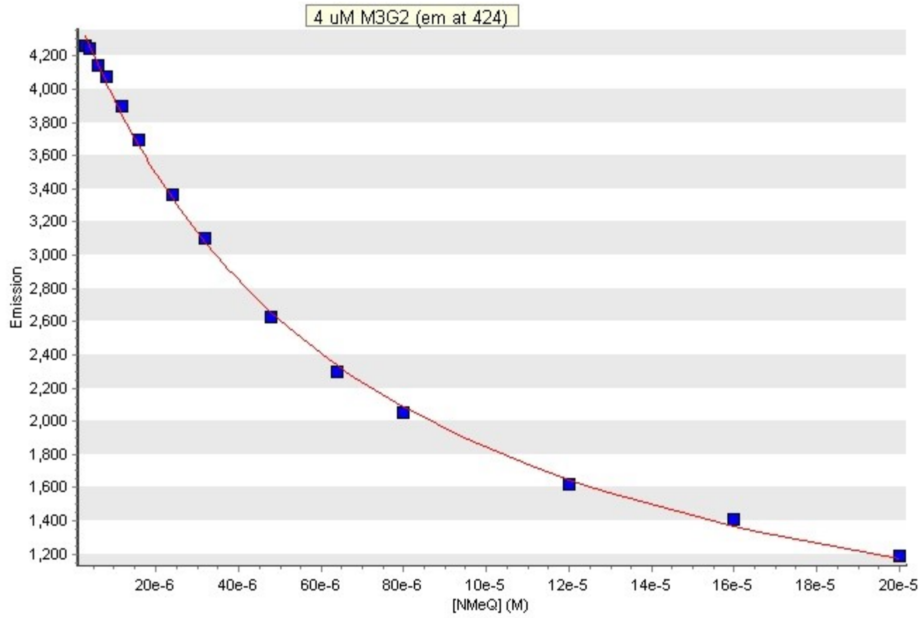

b)

Figure S20. a) Fluorescence spectra from the direct titration of **H2** (4.0 μM) with **NMQ** (0-2000 μM) (Conditions: PBS buffer, pH 7.4, RT); b) Plot of emission intensity at 424 nm (ex. 389 nm) versus [**NMQ**]. The solid curve represents the best non-linear fit of the data to a 1:1 binding model implemented in Scientist™ with $K_a = (1.50 \pm 0.05) \times 10^4\ M^{-1}$.

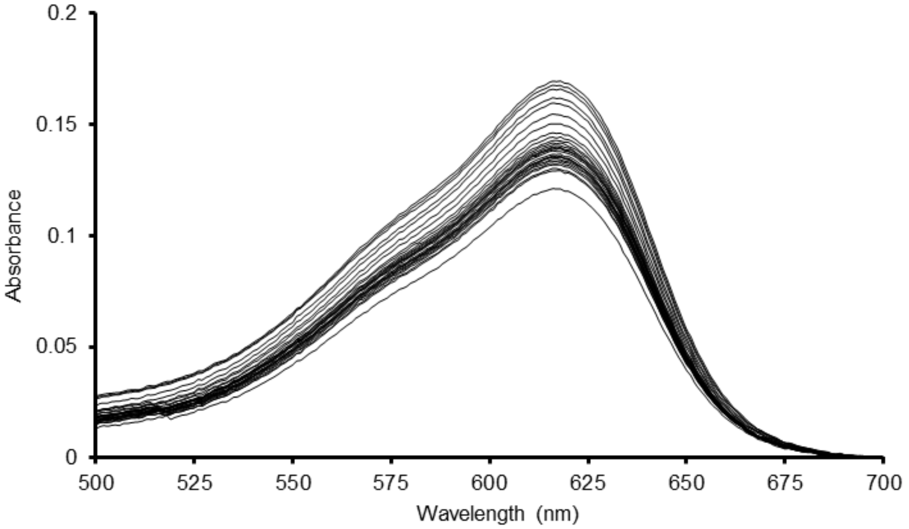

a)

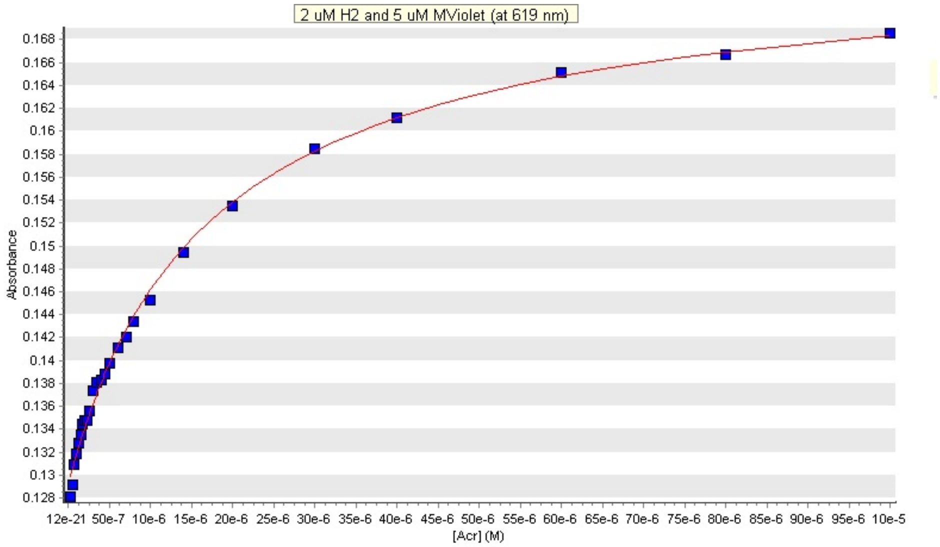

b)

Figure S21. a) UV/Vis spectra from the competitive titration of a mixture of **MViolet** (20.0 μM) and **H2** (2.0 μM) with a solution of **Ac** (0-150 μM) (Conditions: PBS buffer, pH 7.4, RT); b) Plot of absorbance at 619 nm versus [**Ac**]. The solid curve represents the best non-linear fit of the data to a competitive binding model implemented in Scientist^{TM} with $K_a = (5.26 \pm 0.30) \times 10^5$ M^{-1}.

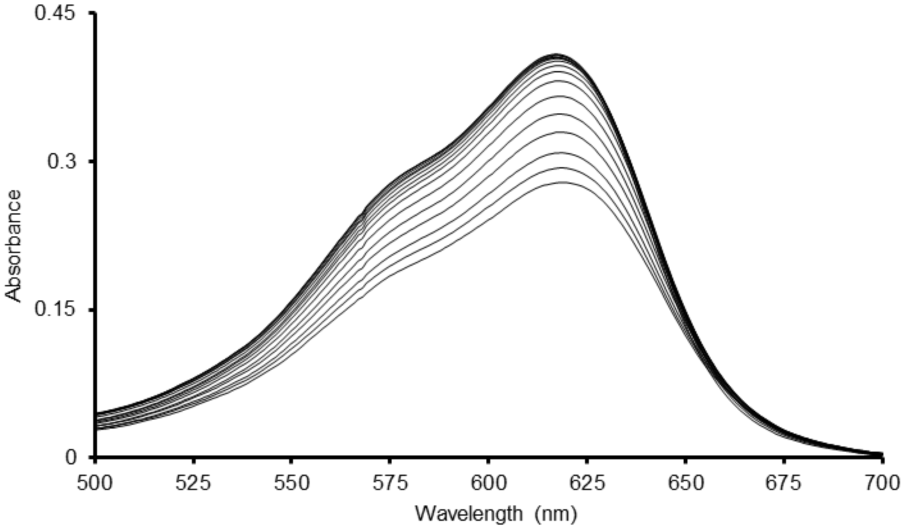

a)

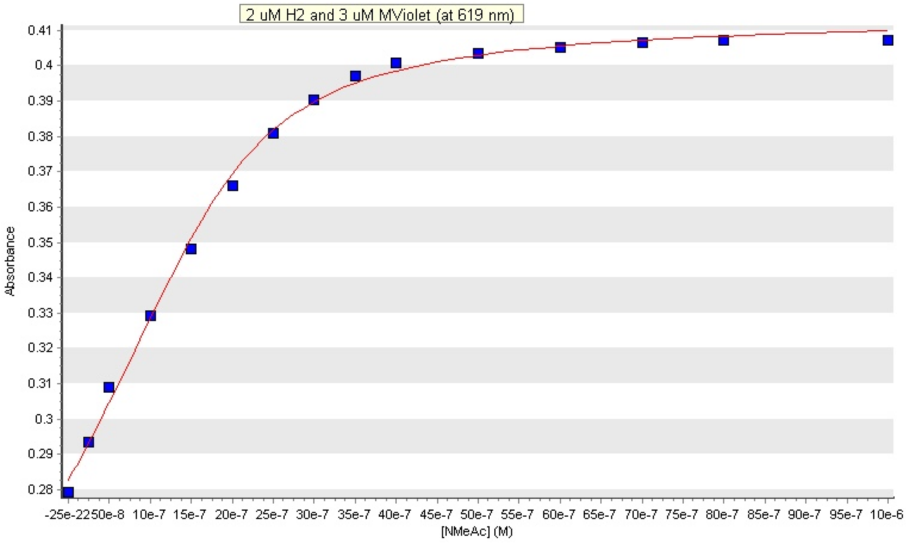

b)

Figure S22. a) UV/Vis spectra from the competitive titration of **MViolet** (3.0 µM) and **H2** (2.0 µM) with **NMeAc** (0-100 µM) (Conditions: PBS buffer, pH 7.4, RT); b) Plot of absorbance at 619 nm versus [**NMeAc**]. The solid curve represents the best non-linear fit of the data to a competitive binding model implemented in Scientist™ with $K_a = (1.07 \pm 0.10) \times 10^7$ M^{-1}.

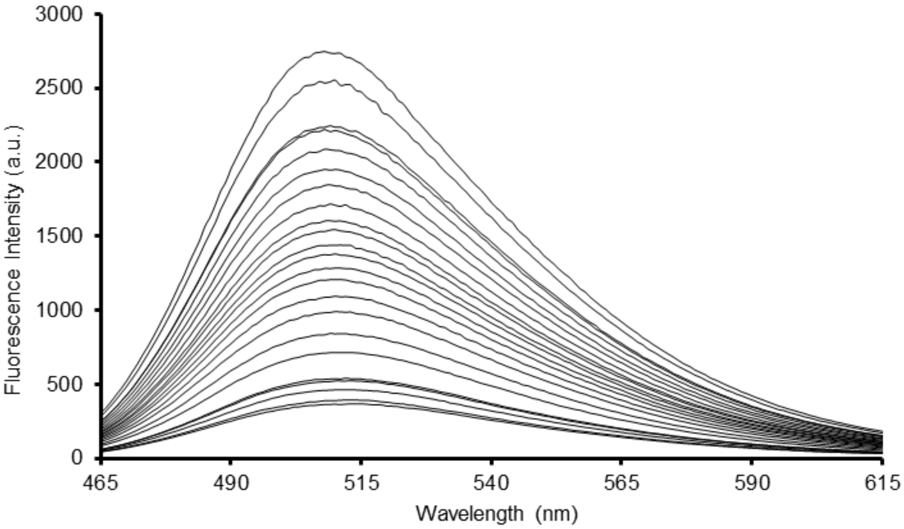

a)

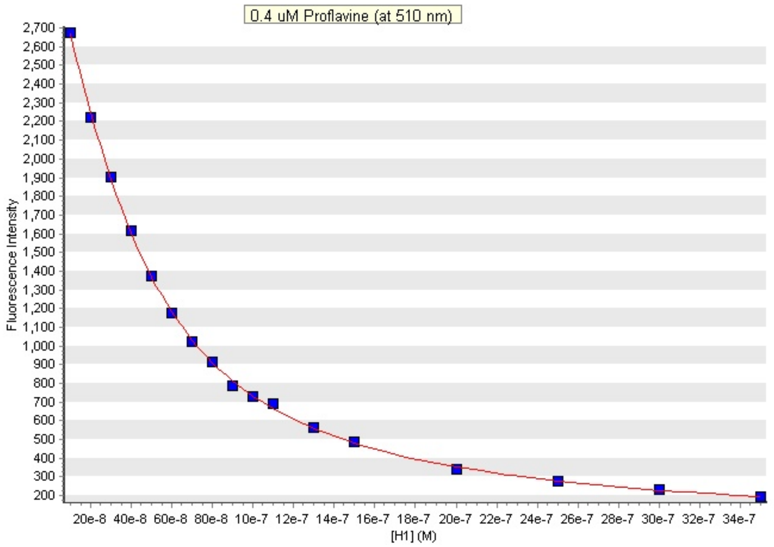

b)

Figure S23. a) Fluorescence spectra from the direct titration of **ProF** (0.4 μM) with **H2** (0-3.5 μM) (Conditions: PBS buffer, pH 7.4, RT); b) Plot of emission intensity at 510 nm (ex. 442 nm) versus [**H2**]. The solid curve represents the best non-linear fit of the data to a 1:1 binding model implemented in Scientist^{TM} with $K_a = (2.78 \pm 0.06) \times 10^6\ M^{-1}$.

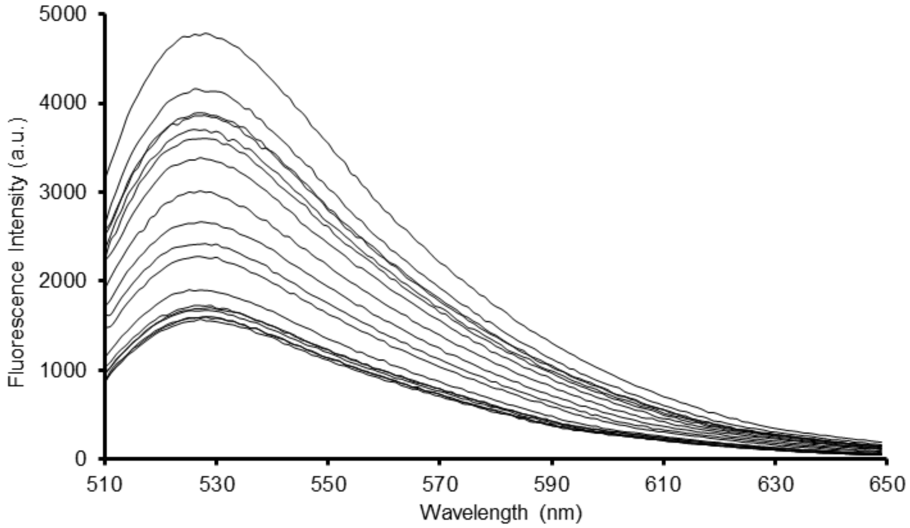

a)

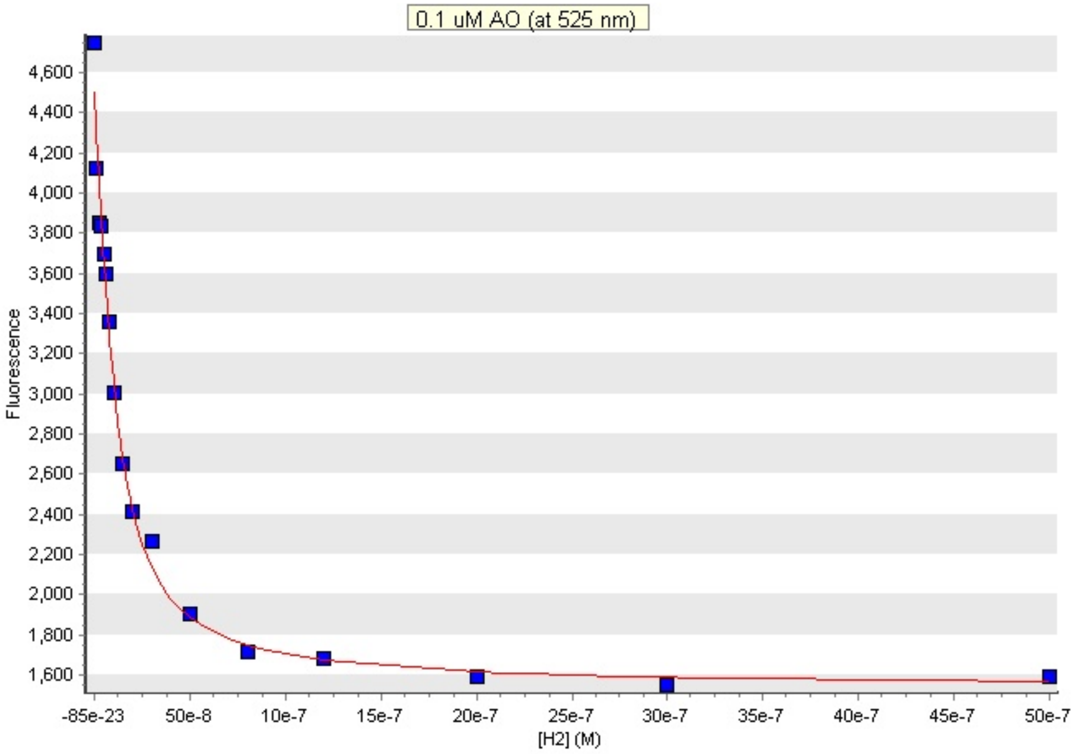

b)

Figure S24. a) Fluorescence spectra from the direct titration of **AcO** (0.1 μM) with **H2** (0-5.0 μM) (Conditions: PBS buffer, pH 7.4, RT); b) plot of the emission intensity at 525 nm versus [**H2**]. The solid curve represents the best non-linear fit of the data to a 1:1 binding model implemented in Scientist™ with $K_a = (1.89 \pm 0.17) \times 10^7\ M^{-1}$.

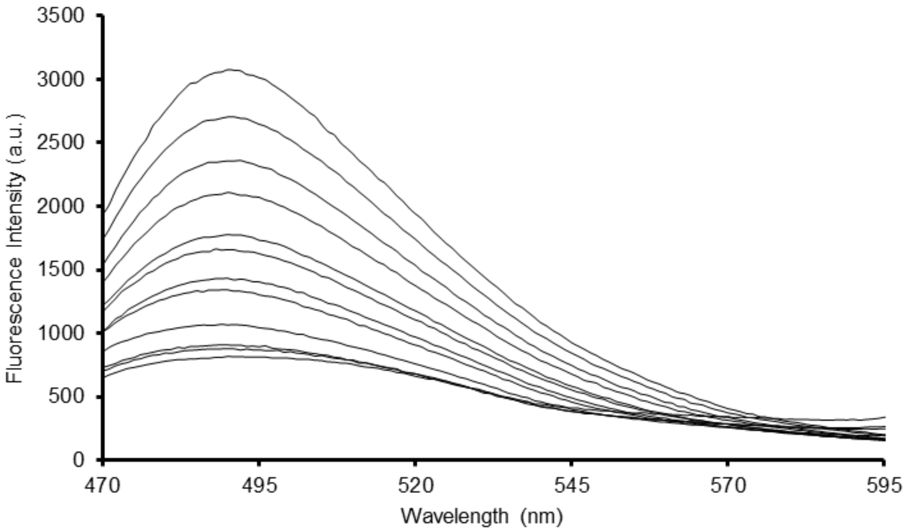

a)

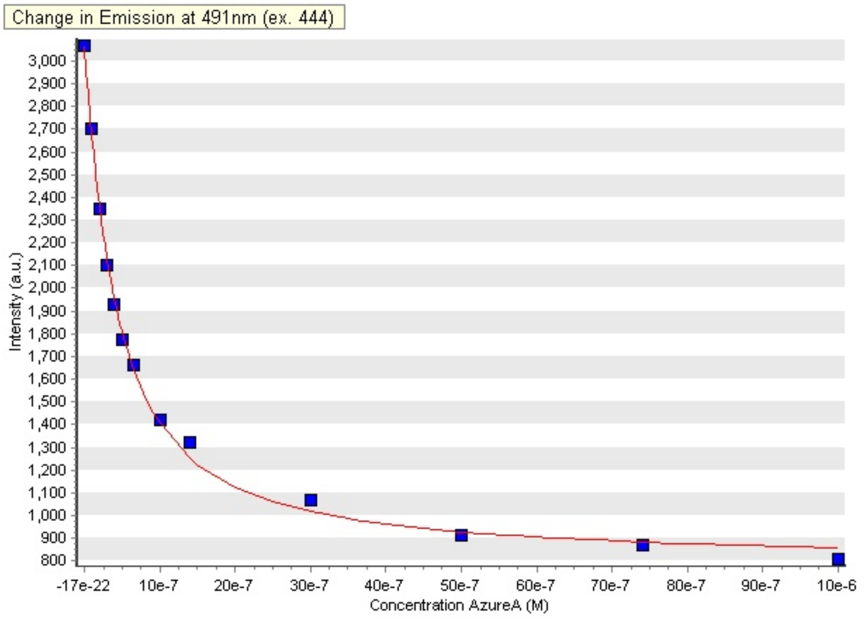

b)

Figure S25. a) Fluorescence spectra from the competitive titration of a mixture of **H2** (0.2 μM) and **ThT** (20.0 μM) with a solution of **AzureA** (0-10.0 μM) (Conditions: PBS buffer, pH 7.4, RT); b) Plot of emission intensity at 491 nm (ex. 444 nm) versus [**AzureA**]. The solid curve represents the best non-linear fit of the data to a competitive binding model implemented in Scientist™ with $K_a = (6.24 \pm 0.53) \times 10^7$ M^{-1}.

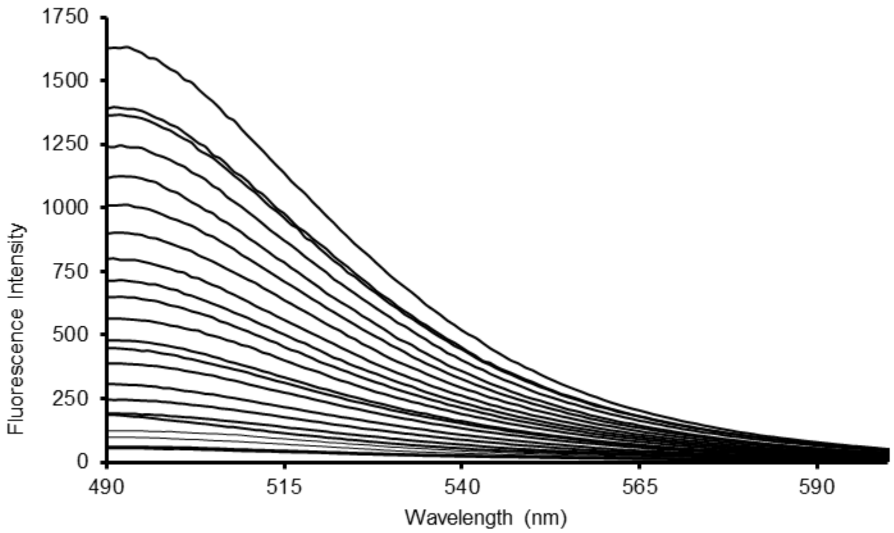

a)

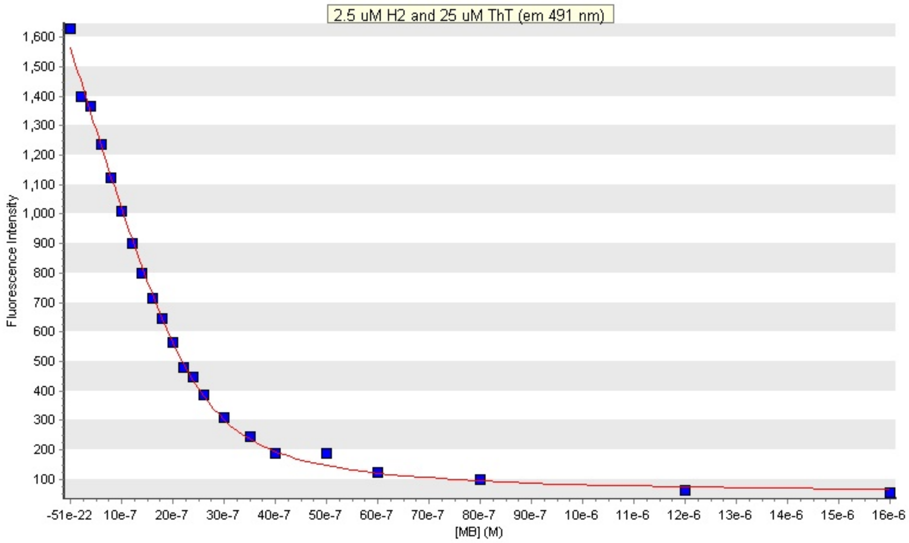

b)

Figure S26. a) Fluorescence spectra from the competitive titration of a mixture of **H2** (2.5 μM) and **ThT** (25.0 μM) with a solution of **MB** (0-16.0 μM) (Conditions: PBS buffer, pH 7.4, RT); b) Plot of emission intensity at 491 nm (ex. 444 nm) versus [**MB**]. The solid curve represents the best non-linear fit of the data to a competitive binding model implemented in Scientist^{TM} with $K_a = (1.24 \pm 0.14) \times 10^8\ M^{-1}$.

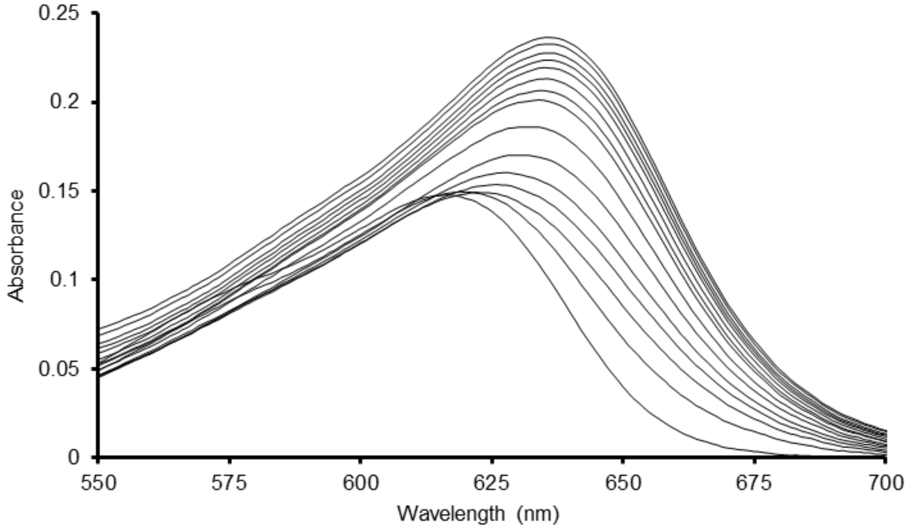

a)

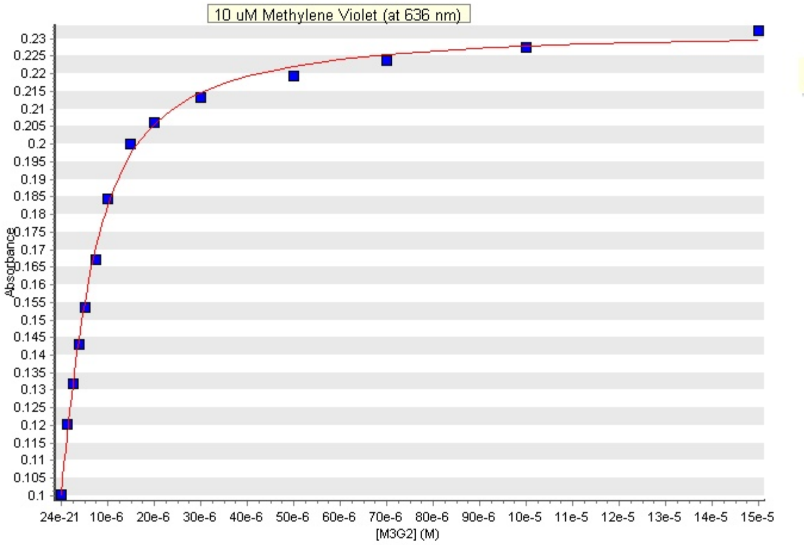

b)

Figure S27. a) UV/Vis spectra from the direct titration of **MViolet** (10.0 μM) with **H2** (0-150.0 μM) (Conditions: PBS buffer, pH 7.4, RT); b) Plot of absorbance at 636 nm versus [**H2**]. The solid curve represents the best non-linear fit of the data to a 1:1 binding model implemented in Scientist™ with $K_a = (2.49 \pm 0.25) \times 10^5\ M^{-1}$.

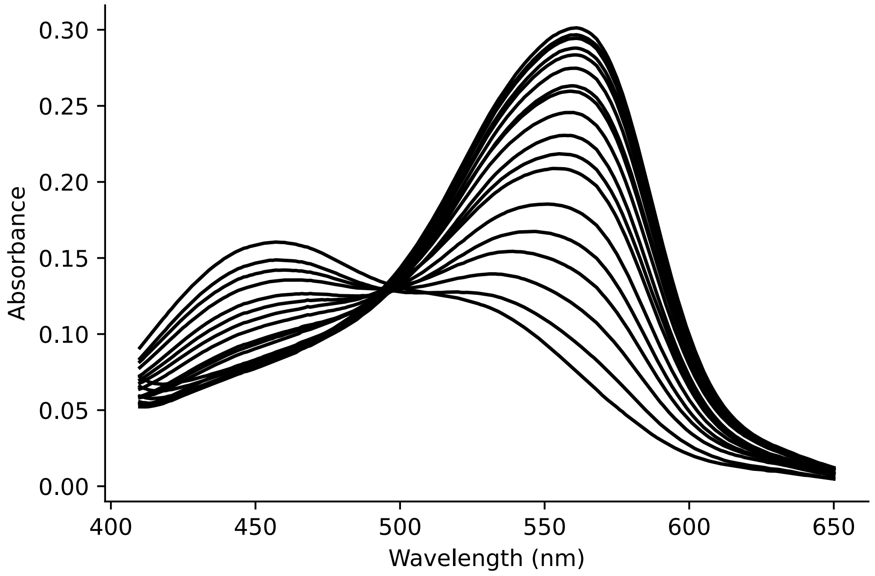

a)

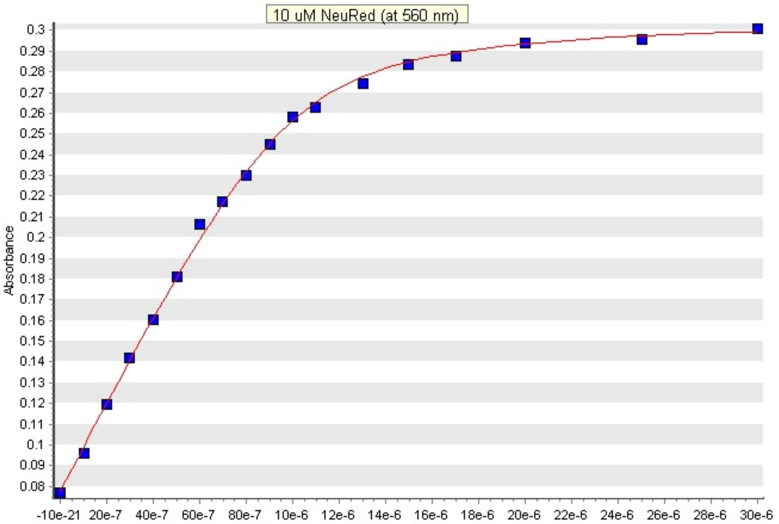

b)

Figure S28. a) UV/Vis spectra from the direct titration of **NeuRed** (10.0 μM) with **H2** (0-30.0 μM) (Conditions: PBS buffer, pH 7.4, RT); b) Plot of absorbance at 560 nm versus [**H2**]. The solid curve represents the best non-linear fit of the data to a 1:1 binding model implemented in Scientist™ with $K_a = (1.95 \pm 0.24) \times 10^6\ M^{-1}$.

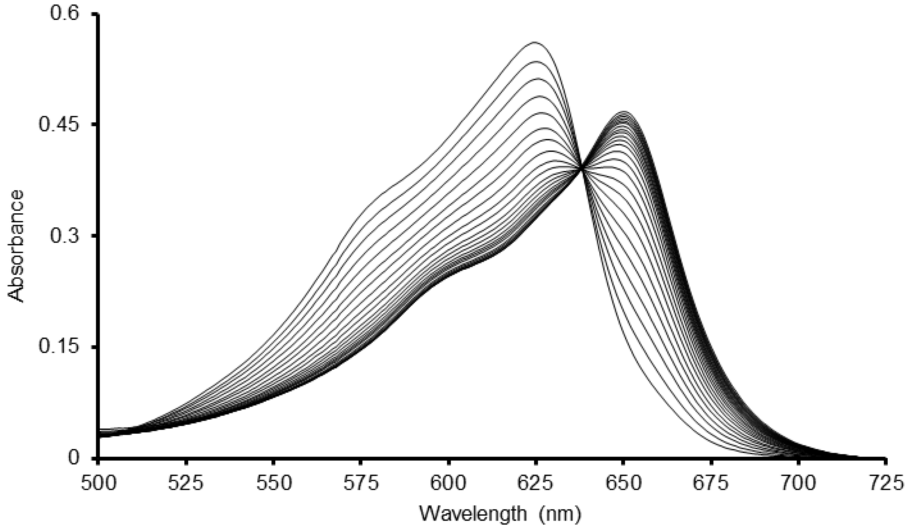

a)

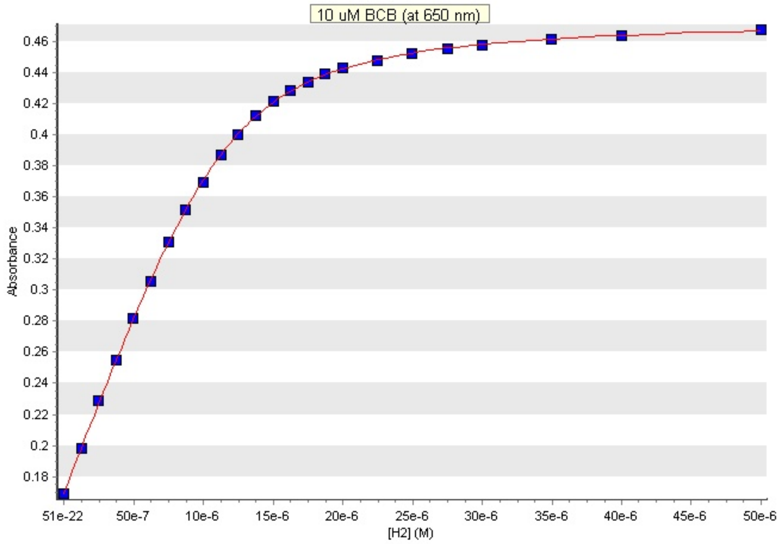

b)

Figure S29. a) UV/Vis spectra from the direct titration of **BCB** (10.0 μM) with **H2** (0-50.0 μM) (Conditions: PBS buffer, pH 7.4, RT); b) Plot of absorbance at 650 nm versus [**H2**]. The solid curve represents the best non-linear fit of the data to a 1:1 binding model implemented in Scientist™ with K_a = $(8.27 \pm 0.15) \times 10^5$ M^{-1}.

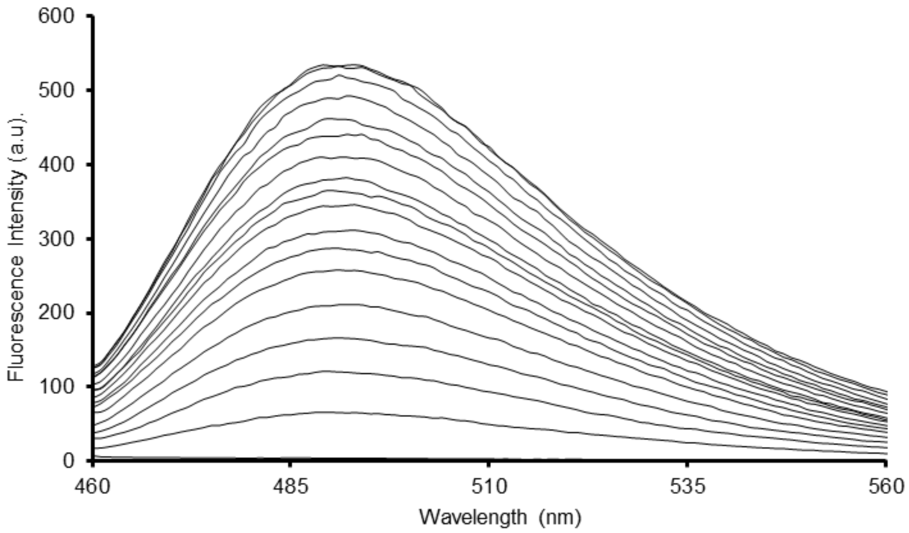

a)

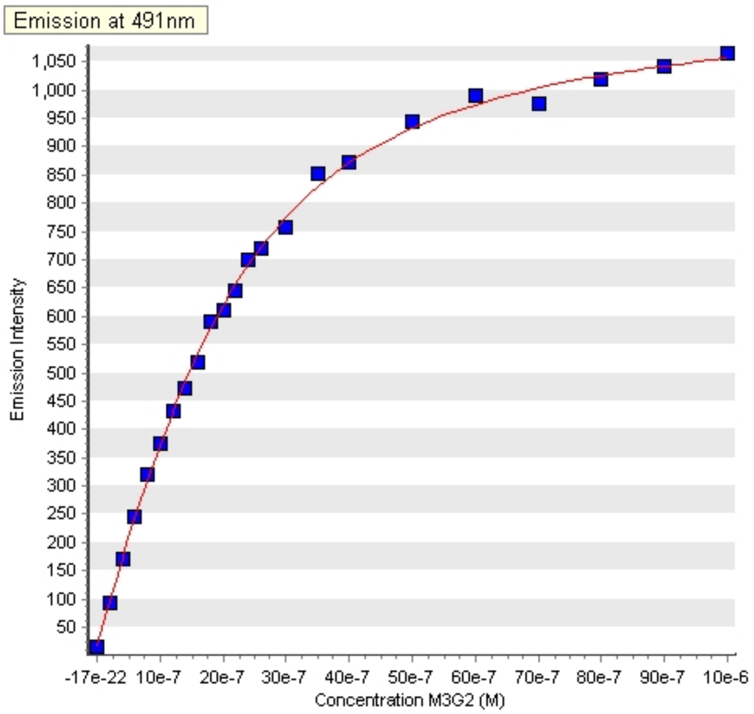

b)

Figure S30. a) Fluorescence spectra recorded during the direct titration of **ThT** (2.0 μM) with **H2** (0-10.0 μM) (Conditions: PBS buffer, pH 7.4, RT); b) Plot of emission intensity at 491 nm (ex. 450 nm) versus [**H2**]. The solid curve represents the best non-linear fit of the data to a 1:1 binding model implemented in Scientist™ with $K_a = (1.15 \pm 0.04) \times 10^6 M^{-1}$.

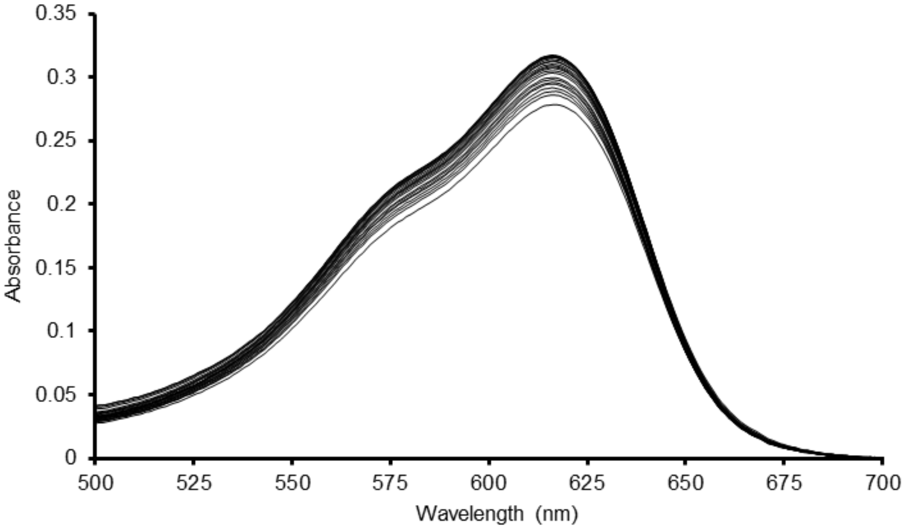

a)

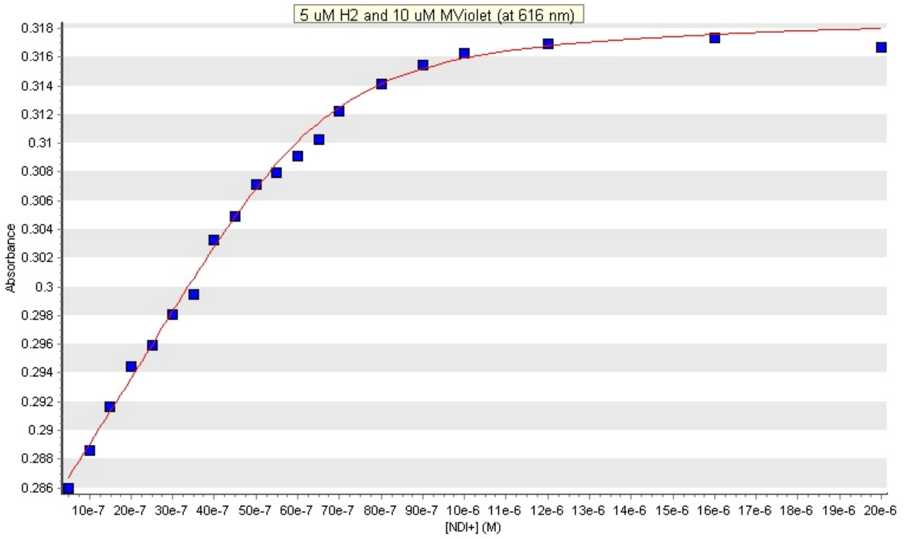

b)

Figure S31. a) UV/Vis spectra from the competitive titration of a mixture of **H2** (5 μM) and **MViolet** (10.0 μM) with a solution of **NDI+** (0-20.0 μM) (Conditions: PBS buffer, pH 7.4, RT); b) Plot of absorbance at 616 versus [**NDI+**]. The solid curve represents the best non-linear fit of the data to a competitive binding model implemented in Scientist™ with $K_a = (1.82 \pm 0.40) \times 10^7\ M^{-1}$.

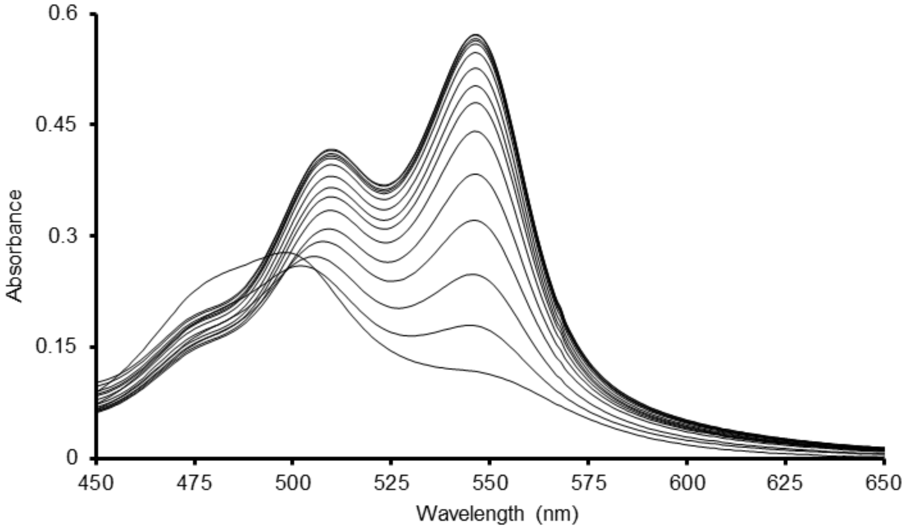

a)

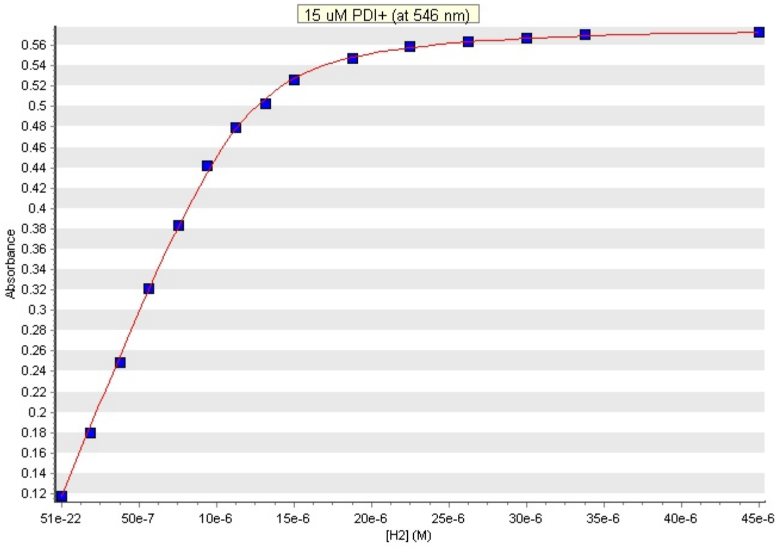

b)

Figure S32. a) UV/Vis spectra from the direct titration of **PDI+** (15 μM) with **H2** (0-45 μM) (Conditions: PBS buffer, pH 7.4, RT); b) Plot of absorbance at 546 versus [**H2**]. The solid curve represents the best non-linear fit of the data to a 1:1 binding model implemented in Scientist^{TM}. K_a was evaluated as $(2.06 \pm 0.13) \times 10^6\ M^{-1}$.

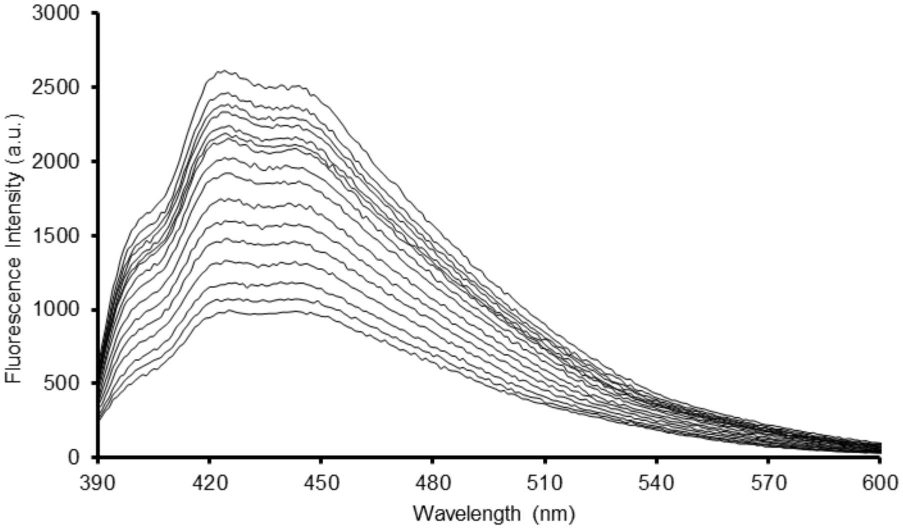

a)

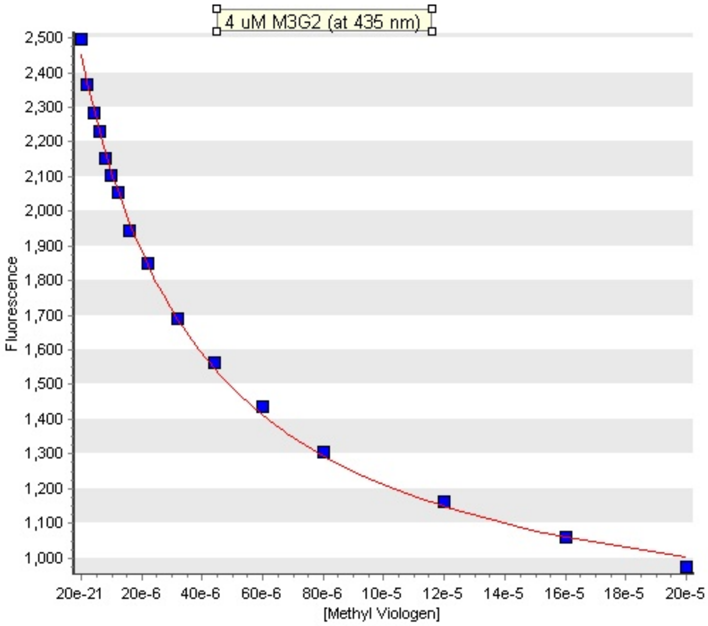

b)

Figure S33. a) Fluorescence spectra from the direct titration of **H2** (4.0 μM) with **Methyl Viologen** (0-200.0 μM) (Conditions: PBS buffer, pH 7.4, RT); b) Plot of emission intensity at 435 nm (ex. 369 nm) versus [**Methyl Viologen**]. The solid curve represents the best non-linear fit of the data to a 1:1 binding model implemented in Scientist™ with $K_a = (2.59 \pm 0.14) \times 10^4\ M^{-1}$.

H2 Dilution Experiment Determination by UV/Vis Spectra

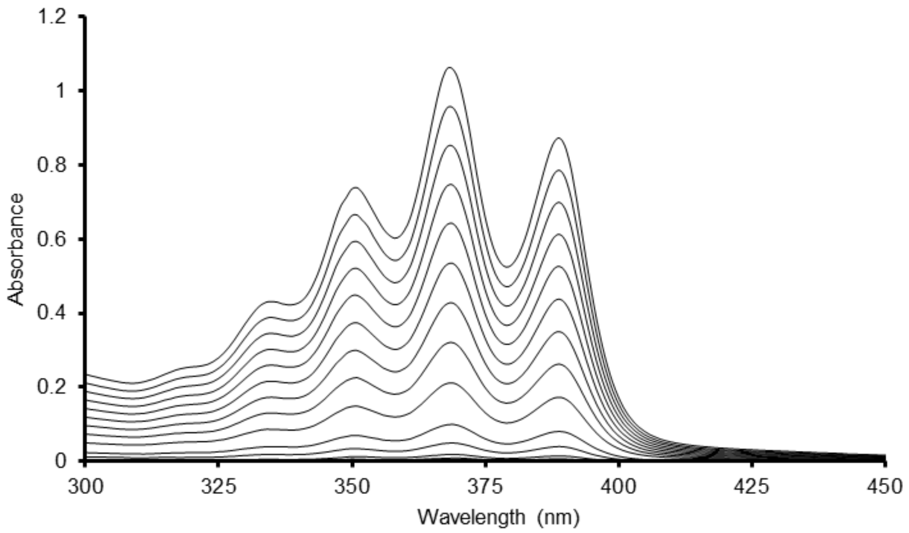

a)

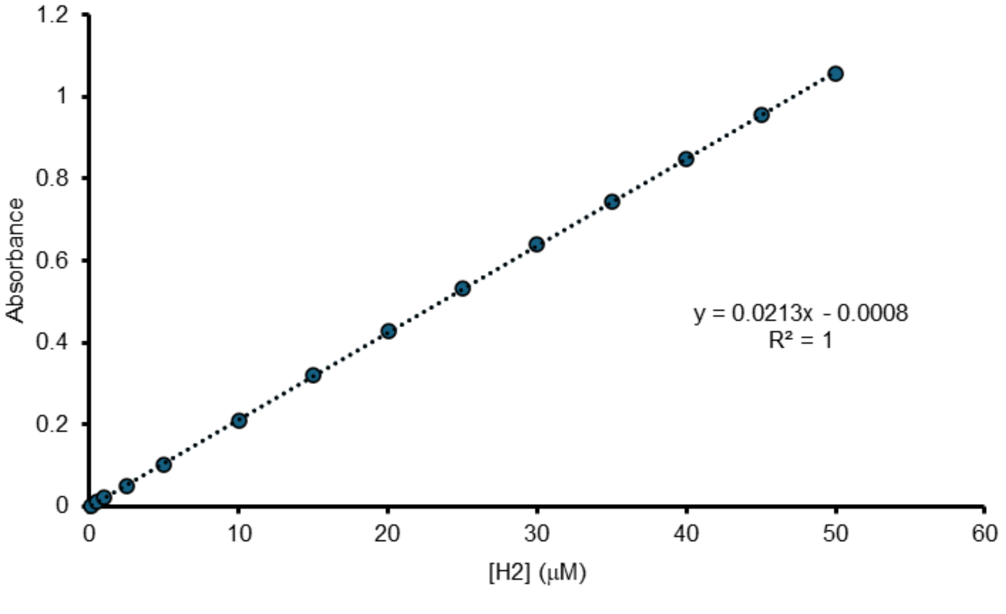

b)

Figure S34. a) UV/Vis spectra recorded for **H2** (0.1-60.0 µM) (HPLC H_2O); b) Plot of emission intensity at 369 nm versus [**H2**], the dotted line represents the best linear fit of the data with the y-intercept set to 0 using the LINEST function in Microsoft Excel. The molar extinction coefficient was evaluated to be 2.12 ± 0.003 × 10^4 M^{-1} cm^{-1}.

^{1}H NMR spectra recorded for selected H2•Guest Complexes

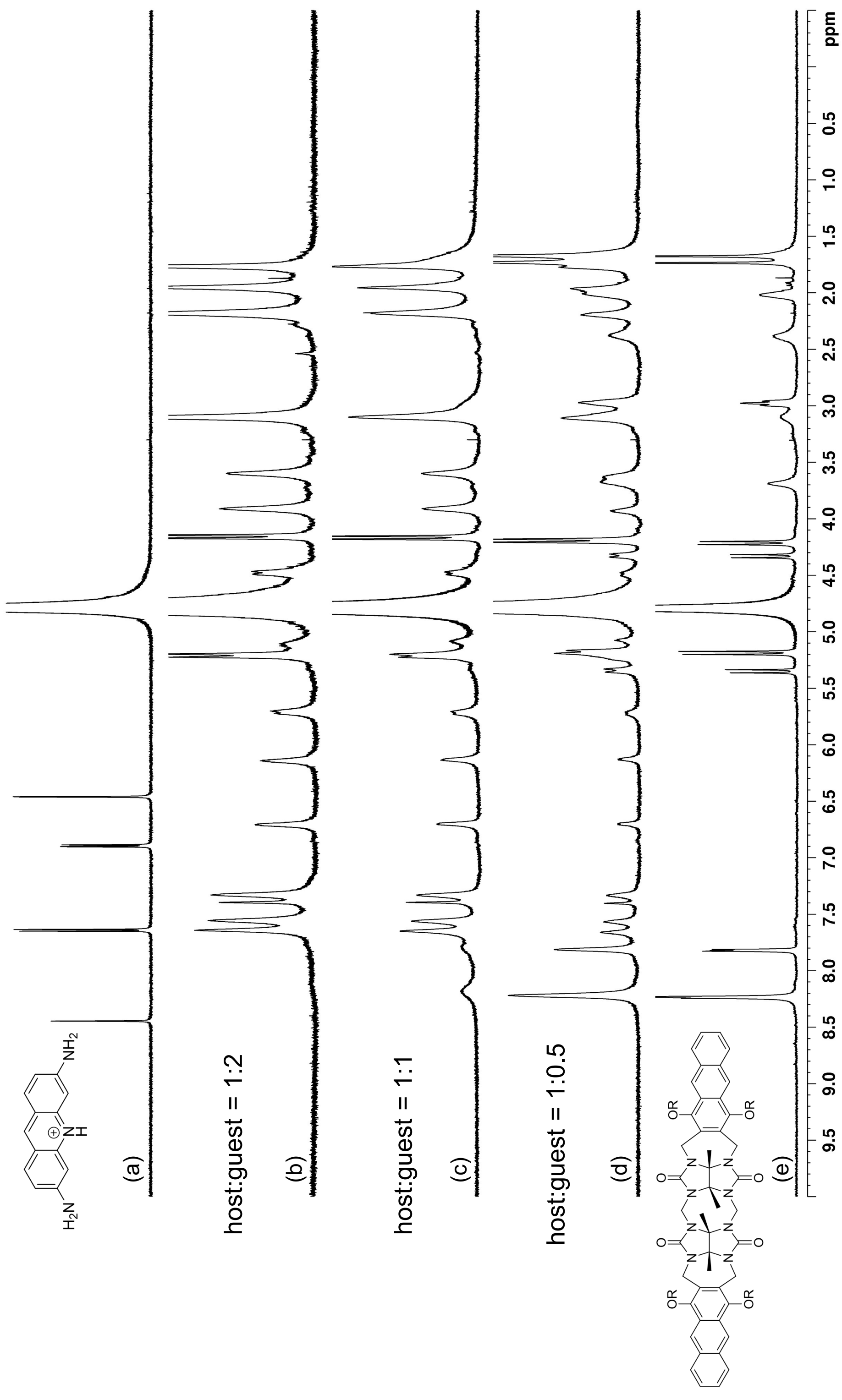

Figure S35. ^{1}H NMR spectra recorded (D_2O, 600 MHz, RT) for a) ProF (0.5 mM), b) a mixture of ProF (1 mM) and **H2** (0.5 mM), c) a mixture of ProF (0.5 mM) and **H2** (0.5 mM), d) a mixture of ProF (0.25 mM) and **H2** (0.5 mM), and e) **H2** (0.5 mM).

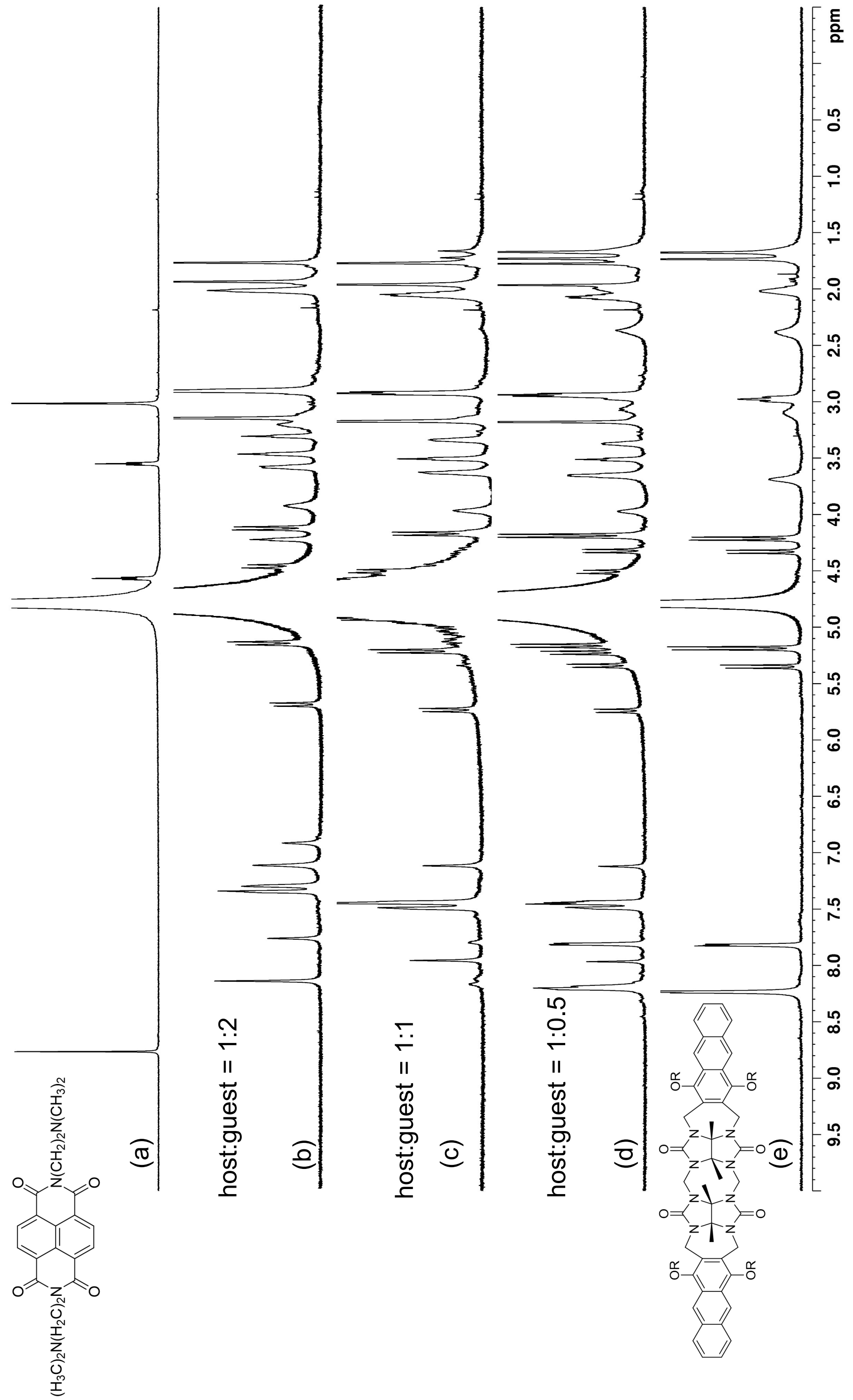

Figure S36. ^{1}H NMR spectra recorded (D_2O, 600 MHz, RT) for a) **NDI+** (0.5 mM), b) a mixture of **NDI+** (1 mM) and **H2** (0.5 mM), c) a mixture of **NDI+** (0.5 mM) and **H2** (0.5 mM), d) a mixture of **NDI+** (0.25 mM) and **H2** (0.5 mM), and e) **H2** (0.5 mM).

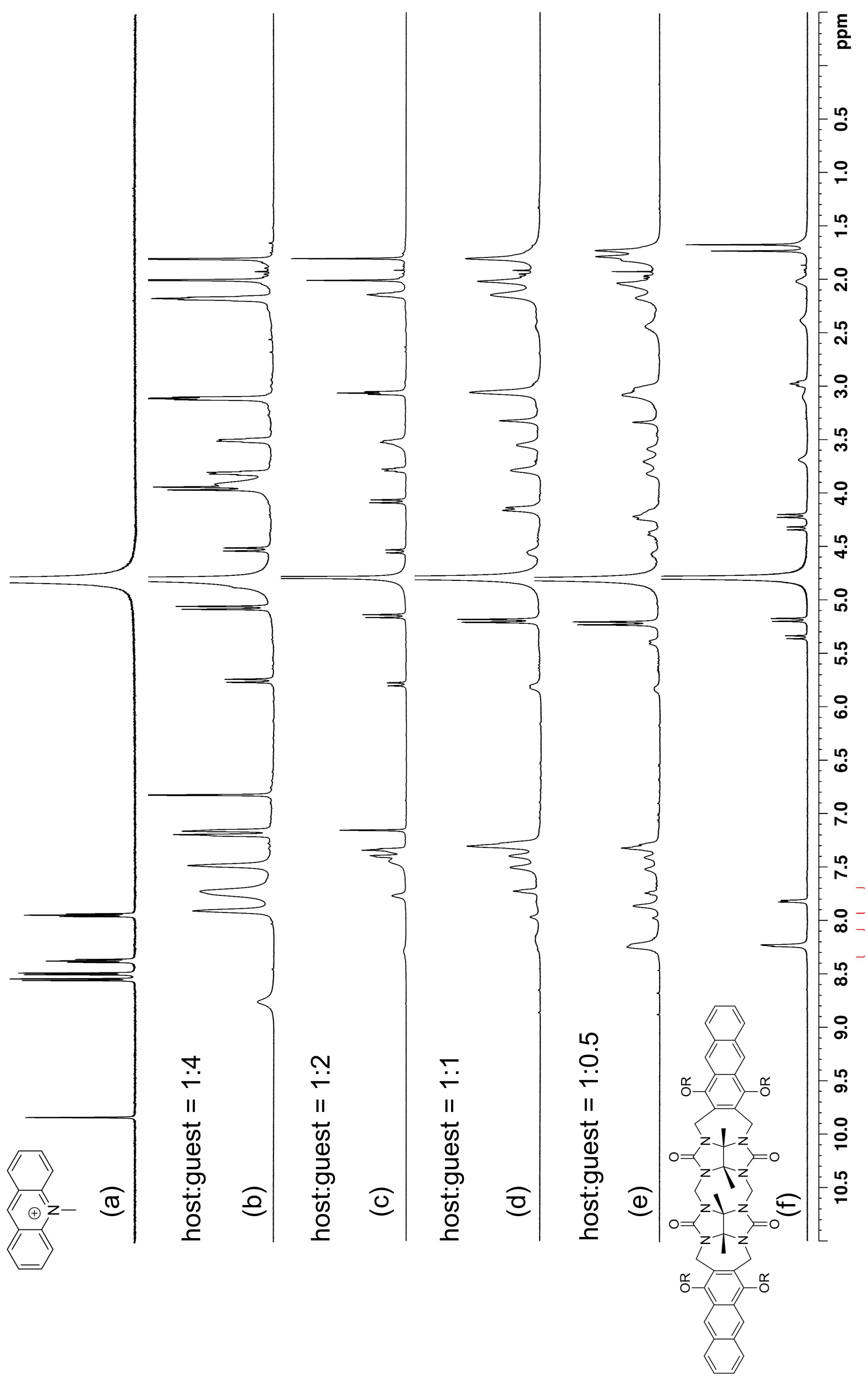

Figure S37. ^{1}H NMR spectra recorded (D_2O, 600 MHz, RT) for a) **NMeAc** (1.0 mM), b) a mixture of **NMeAc** (4.0 mM) and **H2** (1.0 mM), c) a mixture of **NMeAc** (2.0 mM) and **H2** (1.0 mM), d) a mixture of **NMeAc** (1.0 mM) and **H2** (1.0 mM), e) a mixture of **NMeAc** (0.5 mM) and **H2** (1.0 mM), and f) **H2** (1.0mM).

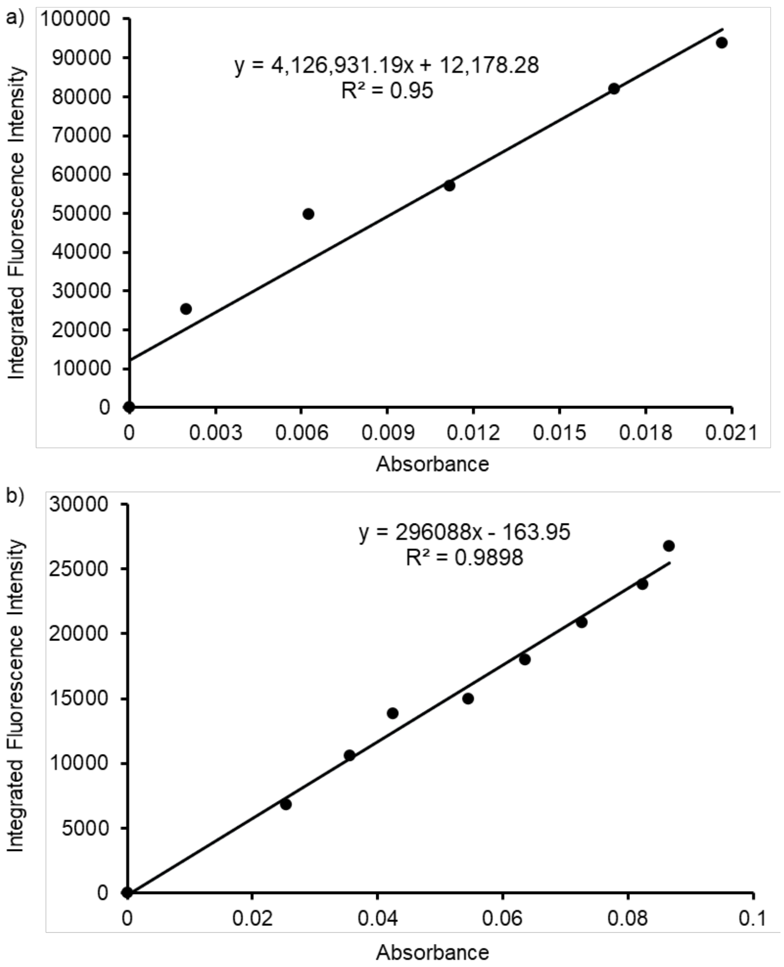

$$\Phi_{\text{sample}} = \Phi_{\text{ref}}\left(\frac{\text{Grad}_{\text{sample}}}{\text{Grad}_{\text{ref}}}\right)\left(\frac{\eta^2{}_{\text{sample}}}{\eta^2{}_{\text{ref}}}\right)$$

(eq. S1)

Where $\text{Grad}_{\text{sample}}$ and Grad_{ref} are the slope of the plot of integrated fluorescence intensity vs absorbance (λ_{ex} = 390 nm) for the sample and reference, respectively, and η_{sample} and η_{sample} are the refractive indices of the solvent used for each solution. **ProF** was used as a reference with known quantum yield (Φ = 0.3433).[S2]

Figure S38. Plot of the integrated fluorescence intensity (from 410 to 800 nm) versus absorbance at 390 nm for aqueous solutions (pure H_2O) of: a) **ProF**, and b) **H2**. The fluorescence quantum yield was calculated using equation S1 given above.

Binding Models Used to Determine Values of K_a with Micromath Scientist™

1:1 Binding Model

```
// Micromath Scientist Model File
// 1:1 Host:Guest binding model
//This model assumes the guest concentration is fixed and host concentration is varied
IndVars: ConcHostTot
DepVars: SpectroscopicSignal
Params: Ka, ConcGuestTot, SpectroscopicSignalMin, SpectroscopicSignalMax
Ka = ConcHostGuest/(ConcHostFree*ConcGuestFree)
ConcHostTot=ConcHostFree + ConcHostGuest
ConcGuestTot=ConcGuestFree + ConcHostGuest
SpectroscopicSignal = SpectroscopicSignalMin + (SpectroscopicSignalMax -
SpectroscopicSignalMin) * (ConcHostGuest/ConcGuestTot)
//Constraints
0 < ConcHostFree < ConcHostTot
0 < Ka
0 < ConcGuestFree < ConcGuestTot
0 < ConcHostGuest < ConcHostTot
```

Competitive Binding Model

```
// MicroMath Scientist Model File
// This model assumes that ConcGtot < ConcHtot.  In the event that ConcHtot < ConcGtot
// then in the Absorb equation ConcGtot should be replaced with ConcHtot
IndVars: ConcAntot
DepVars: Absorb
Params: ConcHtot, ConcGtot, Khg, Kha, AbsorbMax, AbsorbMin
Khg = ConcHG / (ConcH * ConcG)
Kha = ConcHAn / (ConcH * ConcAn)
Absorb = AbsorbMin + (AbsorbMax-AbsorbMin)*(ConcHG/ConcGtot)
ConcHtot = ConcH + ConcHG + ConcHAn
ConcGtot = ConcHG + ConcG
ConcAntot = ConcAn + ConcHAn
0 < ConcHG < ConcHtot
0 < ConcH < ConcHtot
0 < ConcG < ConcGtot
0 < ConcAn < ConcAntot
***
```

pKa Determination Model

```
// Micromath Scientist Model File
IndVars: pH
DepVars: Iobs
```

Params: Imax, Imin, pKa
Iobs=Imax/(1+10^(pH-pKa))+Imin/(1+10^(pKa-pH))

0<pKa<14
0<pH
0<Imax
0<Imin

References Cited

[S1] E. R. Clark, M. J. Ingleson, *Angew. Chem., Int. Ed.* **2014**, *53*, 11306-11309.
[S2] P. Montes-Navajas, A. Corma, and H. Garcia *ChemPhysChem* **2008**, *9*, 713-720.
